# ATM rules neurodevelopment and glutamatergic transmission in the hippocampus but not in the cortex

**DOI:** 10.1038/s41419-022-05038-7

**Published:** 2022-07-16

**Authors:** Elisa Focchi, Clara Cambria, Lara Pizzamiglio, Luca Murru, Silvia Pelucchi, Laura D’Andrea, Silvano Piazza, Lorenzo Mattioni, Maria Passafaro, Elena Marcello, Giovanni Provenzano, Flavia Antonucci

**Affiliations:** 1 Department of Medical Biotechnology and Translational Medicine (BIOMETRA), University of Milan, Milano, Italy; 2 Department de Biology, Ecole Normale Supérieure, Paris, France; 3 Institute of Neuroscience, IN-CNR, Milano, Italy; 4 NeuroMI Milan Center for Neuroscience, Università Milano-Bicocca, Milan, 20126 Italy; 5 Department of Pharmacological and Biomolecular Sciences, University of Milan, Milano, Italy; 6 Computational Biology, International Centre for Genetic Engineering and Biotechnology, Trieste, Italy; 7 Department of Cellular, Computational and Integrative Biology - CIBIO, University of Trento, Trento, Italy

**Keywords:** Cellular neuroscience, Neurodevelopmental disorders

## Abstract

Interest in the function of ataxia-telangiectasia-mutated protein (ATM) is extensively growing as evidenced by preclinical studies that continuously link ATM with new intracellular pathways. Here, we exploited *Atm*^+/−^ and *Atm*^−/−^ mice and demonstrate that cognitive defects are rescued by the delivery of the antidepressant Fluoxetine (Fluox). Fluox increases levels of the chloride intruder NKCC1 exclusively at hippocampal level suggesting an ATM context-specificity. A deeper investigation of synaptic composition unveils increased Gluk-1 and Gluk-5 subunit-containing kainate receptors (KARs) levels in the hippocampus, but not in the cortex, of *Atm*^+/−^ and *Atm*^−/−^ mice. Analysis of postsynaptic fractions and confocal studies indicates that KARs are presynaptic while in vitro and ex vivo electrophysiology that are fully active. These changes are (i) linked to KCC2 activity, as the KCC2 blockade in *Atm*^+/−^ developing neurons results in reduced KARs levels and (ii) developmental regulated. Indeed, the pharmacological inhibition of ATM kinase in adults produces different changes as identified by RNA-seq investigation. Our data display how ATM affects both inhibitory and excitatory neurotransmission, extending its role to a variety of neurological and psychiatric disorders.

## Introduction

Ataxia-telangiectasia mutated (ATM) is a large kinase known as DNA repair protein [1–4]. Whereas in proliferating cells ATM acts as fundamental regulator of the cell fate [5–7], in postmitotic neurons a complete description of its activities is still missing. Mutations in the gene encoding for ATM cause a rare multisystemic disease known as ataxia-telangiectasia (A-T), characterized by a severe phenotype [8]. A-T patients display progressive ataxia and motor abnormalities but also mild to moderate intellectual impairments which can affect different domains, such as language, memory, and executive functions [7, 9]. Coherently, ATM participates to (i) synaptic vesicles endocytosis in cortical neurons [10–12], (ii) cytoplasmatic calcium homoeostasis in hippocampal cells [13], (iii) autophagy-lysosomal pathway [14, 15], and (iv) GABAergic development in the hippocampus [16]. Here, starting from the pharmacological evidence that the control of cognitive defects in *Atm* heterozygous (*Atm*^+/−^) and knockout (*Atm*^−/−^) mice is achieved by delivering the selective serotonin reuptake inhibitor Fluoxetine (Fluox), we demonstrate that ATM differentially regulates hippocampal and cortical development as well as synaptic properties.

We showed that in the hippocampus ATM rules development of inhibitory system [15] via a KCC2-dependent mechanism and now that it modulates also the excitatory synapse. The glutamatergic synapse displays normal glutamate release in basal condition but increased excitatory currents under evoked stimulation. This arises from the higher expression of Gluk-5- and Gluk-1-subunits containing kainate receptors (KARs) at presynaptic site. These modifications are KCC2-dependent and developmentally regulated, indeed the acute blockade of ATM activity in adult wt mice produces different changes as indicated by RNA seq investigation. Data here presented indicate how ATM affects excitatory neurotransmission extending the role of ATM to a much wider spectrum of neurological and psychiatric disorders.

## Results

### Cognitive disabilities in *Atm*^+/−^ mice are reverted by prenatal and postnatal Fluoxetine treatments

**Fig. 1:**
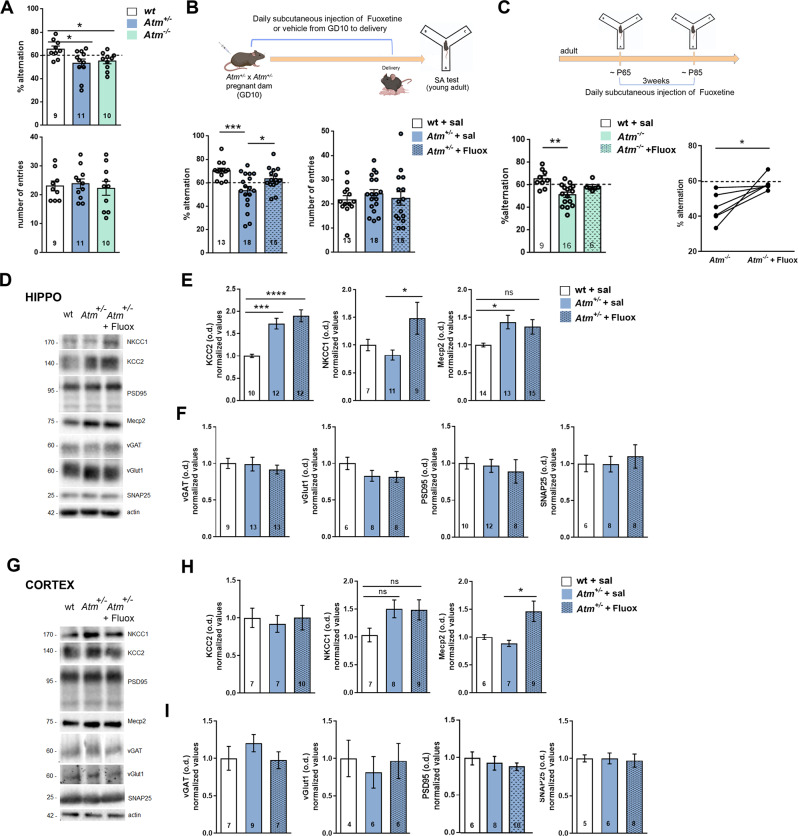
Prenatal and postnatal fluoxetine (Fluox) treatments revert cognitive disabilities in *Atm*^+/−^ and *Atm*^−/−^ mice. **A** Percentage of alternation (top) and number of entries (bottom) in the Spontaneous Alternation (SA) test in wt, *Atm*^+/−^ *and Atm*^−/−^ P40 mice. Ordinary one-way ANOVA followed by Dunnett’s multiple comparison test; *p < 0.05. **B** Prenatal Fluox treatment (top). We mated *Atm*^+/−^ female with *Atm*^+/−^ males and injected Fluox and starting from embryonic day 10 to delivery, we daily injected subcutaneously pregnant *Atm*^+/−^ dams with Fluox (10 mg/kg). In this first protocol, no *Atm*^−/−^ mice were born, maybe due to the embryonic fragility linked to the complete ATM absence. Percentage of alternation and number of entries (bottom) in the SA test in wt + sal, *Atm*^+/−^ + sal and in *Atm*^+/−^ + Fluox. Ordinary one-way ANOVA followed by Tukey’s multiple comparison test; *p < 0.05, ***p < 0.001. **C** Fluoxetine treatment in adult mice (top). *Atm*^−/−^ mice shows rescued % of alternation when chronically treated with Fluox for three weeks. Kruskal–Wallis test followed by Dunn’s multiple comparison test, **p < 0.01, Wilcoxon paired test; *p* = 0.031. **D** Representative Western blot lanes for hippocampal samples. **E** Western blotting analysis shows increased KCC2 and Mecp2 expression in the hippocampus of *Atm*^+/−^ mice but no rescue was observed in *Atm*^+/−^ mice prenatally treated with Fluox; ordinary one-way ANOVA followed by Tuckey’s multiple comparison test; *p < 0.01, ***p < 0.001, ****p < 0.0001. Fluox treatment promotes NKCC1 expression specifically; ordinary one-way ANOVA followed by Tuckey’s multiple comparison test; *p < 0.01. **F** No difference was observed in synaptic markers among wt and *Atm*^+/−^ mice in the hippocampus, not even upon Fluox treatment. **G** Representative Western blot lanes for cortical samples. **H** KCC2, NKCC1, and MeCP2 protein levels are not affected in the cortex of *Atm*^+/−^ mice, not even upon Fluox administration. **I** No difference was observed in synaptic markers among wt and *Atm*^+/−^ in the cortex. Numbers in bars indicate the number of animals. ns means not significant^.^ Bars in graphs indicate ±SEM.

Previous studies reported cognitive abnormalities in *Atm*^−/−^ adult mice [17] and impaired neuronal plasticity in hippocampal slices prepared from *Atm*^−/−^ animals [11, 18]. Here, to investigate the direct impact of ATM on spatial working memory we tested wt and both symptomatic *Atm*^−/−^ and asymptomatic *Atm*^+/−^ young-adult (P40) mice for the low-stressful task Y-maze spontaneous alternation (SA, [19]). As indicated in Fig. 1A, *Atm*^+/−^ and *Atm*^−/−^ mice exhibit a significant reduction in the percentage of spontaneous alternations. To promote the functional recovery, we analysed effects induced by the antidepressant Fluoxetine (Fluox) as it is able to interfere not only with the serotoninergic system but also with the GABAergic transmission [20]. We exploited the prenatal exposure to Fluox in pregnant *Atm*^+/−^ female (from GD10 to delivery, as in ref. [21]). We evaluated cognitive functions in P40 *Atm*^+/−^ offspring treated with Fluox compared to P40 *Atm*^+/−^ animals born from *Atm*^+/−^ dams treated with subcutaneous saline solution and to P40 wt generated by wt pregnant females treated with saline solution (Fig. 1B, see the scheme). As shown in Fig. 1B, our approach was able to fully rescue the cognitive function in the SA task in *Atm*^+/−^ offspring. Since no *Atm*^−/−^ mice were born upon this protocol (maybe due to the embryonic fragility linked to the complete ATM absence), we directly treated *Atm*^−/−^ adult animals with subcutaneous Fluox injections for 3 weeks. We found that whereas this approach was effective in restoring SA defects in *Atm*^−/−^ mice (Fig. 1C) it was not able to correct impairments highlighted by Novel Object Recognition (NOR) test (Suppl Fig. 1A). Of note, the non-spatial learning and association task NOR test requires functionality of hippocampus, but also of perirhinal cortex [22] and para-hippocampal regions [23]. Thus, Fluoxetine treatment corrects spatial deficits in *Atm*^+/−^ and *Atm*^−/−^ mice, leaving unchanged the non-spatial learning impairments.

Then, we explanted brain tissues from mice that received Fluox (or saline) during prenatal life for the biochemical and confocal characterizations. In the hippocampus, we highlighted increments in the marker for inhibitory synapses vGAT by the confocal analysis (Supplementary Fig. 1B), whereas by biochemical approach no changes in synaptic proteins appeared between wt and *Atm*^+/−^ mice (Fig. 1D–F). As expected, in *Atm*^+/−^ animals we found alterations in proteins linked to GABAergic development as showed by the higher expression of chloride extruder KCC2 and the epigenetic regulator MeCP2 (Fig. 1D, E). Here, Fluox prenatal delivery did not normalize neither KCC2 nor MeCP2 levels or any other pre- and postsynaptic proteins analysed (Fig. 1F). Interestingly, Fluox promoted the expression of the chloride intruder NKCC1 (Fig. 1E), which also mediates GABA hyperpolarizing effects [24]. Surprisingly, analyses of similar markers in the cortex of *Atm*^+/−^ mice compared to wt (Fig. 1F) did not reveal changes in KCC2, NKCC1 or MeCP2 levels and accordingly prenatal Fluox treatment results completely ineffective (Fig. 1G–I) in this brain region.

Thus, prenatal Fluox administration ameliorates cognitive defects in *Atm*^+/−^ animals and increases NKCC1 levels specifically in the hippocampus. Unexpectedly, our biochemical results suggest that ATM differently affects neurons in hippocampus and cortex, reason why we decided to further investigate this context diversity.

### The excitatory-to-inhibitory GABA switch is normal in *Atm*^+/−^ and *Atm*^−/−^ cortical neurons

**Fig. 2:**
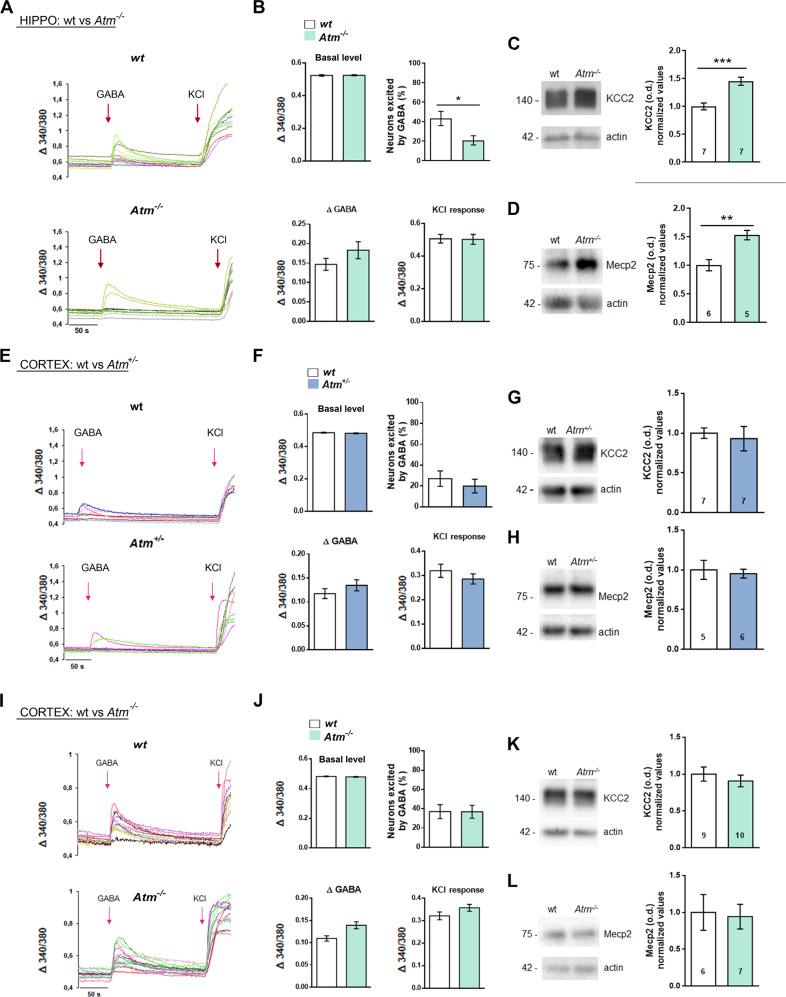
ATM does not control the excitatory-to-inhibitory GABA switch in cortical neurons. **A** Representative traces of calcium transients in 6DIV wt and *Atm*^−/−^ hippocampal neurons. **B** No differences in basal intracellular calcium levels are detected in wt vs *Atm*^−/−^. The number of GABA-responding neurons is reduced in *Atm*^−/−^ hippocampal neurons; t test: *p* = 0.013. Calcium increments induced by exogenous GABA (Δ GABA) are comparable in wt and *Atm*^−/−^. Upon KCl stimulus no variations were measured in the generated calcium fluxes among the two groups. (Coverslips: wt = 4 vs. *Atm*^−/−^ = 5; total cells: wt = 156 vs. *Atm*^−/−^ = 182; independent experiments = 3. **C** and **D** KCC2 and Mecp2 protein levels are increased in the hippocampi of P40 *Atm*^−/−^ mice respect to wt controls. KCC2 *t* test: *p* = 0.0005; Mecp2 *t* test: *p* = 0.0032. **E** Representative traces of calcium transients in 6DIV wt and *Atm*^+/−^ cortical neurons. **F** Basal calcium levels and percentage of GABA-responding neurons are not altered in wt vs. *Atm*^+/−^, neither are calcium increments upon GABA and KCl administration. Coverslips: wt = 3 vs. *Atm*^+/−^ = 3; total cells: wt = 101 vs. *Atm*^+/−^ = 92; independent experiments = 3. **G** and **H** KCC2 and Mecp2 protein levels are unaltered in P40 *Atm*^+/−^ cortices respect to wt. **I** Representative traces of calcium transients in 6DIV wt and *Atm*^−/−^ cortical neurons. **J** Basal calcium levels and percentage of GABA-responding neurons are not altered in wt vs. *Atm*^−/−^, neither are calcium increments upon GABA and KCl administration. Coverslips: wt = 5 vs. *Atm*^−/−^ = 6; total cells: wt = 220 vs. *Atm*^−/−^ = 242; independent experiments = 3. **K** and **L** KCC2 and Mecp2 protein levels are unaltered in P40 *Atm*^−/−^ cortices respect to wt. Numbers in bars indicate the number of animals. Bars in graphs indicate ±SEM.

Since we described a premature excitatory-to-inhibitory GABA switch in *Atm*^+/−^ hippocampal neurons [15], we challenged hippocampal *Atm*^−/−^ cells in calcium imaging technique. In 6 div wt and *Atm*^−/−^ hippocampal cultures no differences were detected in terms of basal level of intracellular calcium (Fig. 2A, B) and calcium increments induced by exogenous GABA (100 µM, Fig. 2A, B, Δ GABA), suggesting a similar expression of GABA-A receptors. As expected, we found a premature GABA inhibitory effect in *Atm*^−/−^ neurons indicated by the lower percentage of cells excited by GABA (Fig. 2A, B). Since levels of voltage operated calcium channels (VOCCs) may influence these results, we applied 50 mM KCl to wt and *Atm*^−/−^ hippocampal cultures and no variations were measured in calcium fluxes between the two groups (Fig. 2A, B), suggesting normal VOCCs expression. Investigation of KCC2 and MeCP2 levels in hippocampal tissues from P40 mice revealed increased expression of these two proteins in the *Atm*^−/−^ group (Fig. 2C, D). Similar results in terms of KCC2 levels have been collected in hippocampi of P12–13 *Atm*^−/−^ mice, confirming alterations in the GABAergic development at earlier time points (Supplementary Fig. 2A).

Then, we performed similar experiments in *Atm*^+/−^ and *Atm*^−/−^ cortical neurons (Fig. 2E, F and I, J). Analysis of basal calcium levels and calcium transients induced by the exogenous GABA were similar in *Atm*^+/−^ and *Atm*^−/−^ cells respect to wt cortical cultures (Fig. 2F–J and I, J). Conversely to what described above, percentage of *Atm*^+/−^ and *Atm*^−/−^ neurons responding to GABA with neuronal excitation was similar to that of wt group (Fig. 2F–J and I, J) as well as comparable calcium transients induced by KCl (Fig. 2F–J and I, J). Accordingly, analysis of KCC2 and MeCP2 expression in cortical tissues from *Atm*^+/−^ (Fig. 2G, H) or *Atm*^−/−^ (Fig. 2K, L) vs. wt P40 mice revealed no differences.

These data indicate that in cortical neurons ATM does not control neither KCC2 expression levels nor GABAergic development.

### ATM deficiency affects basal synaptic transmission in hippocampal but not in cortical neurons

**Fig. 3:**
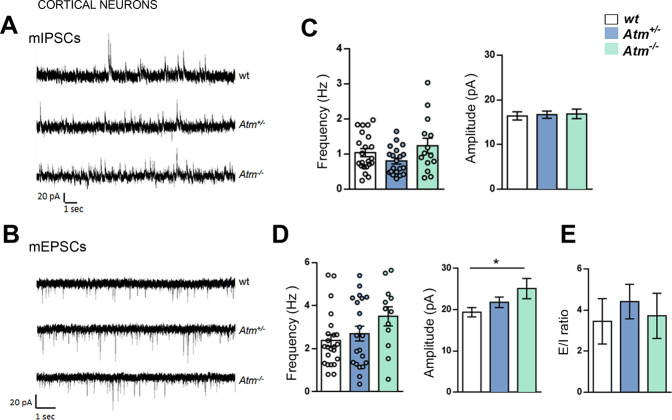
ATM deficiency affects basal synaptic transmission in hippocampal but not in cortical neurons. **A** and **B** Representative traces of mIPSCs and mEPSCs recorded in 13–14 DIV cortical neurons obtained from wt, *Atm*^+/−^ and *Atm*^−/−^ mouse embryos. **C** Analysis of inhibitory frequency and amplitude (mIPSCs frequency: ordinary one-way ANOVA followed by Holm–Sidak’s multiple comparison test ns, wt = 22, *Atm*^+/−^ = 20, *Atm*^−/−^ = 14; mIPSCs amplitude: Kruskal–Wallis test followed by Dunn’s multiple comparison test ns, wt = 22, *Atm*^+/−^ = 21, *Atm*^−/−^ = 14; independent experiments = 3) from cultured neurons. **D** Analysis of excitatory frequency and amplitude (mEPSCs frequency: Kruskal–Wallis test followed by Dunn’s multiple comparison test ns, wt = 23, *Atm*^+/−^ = 21, *Atm*^−/−^ = 12; mEPSCs amplitude: ordinary one-way ANOVA followed by Tukey’s multiple comparison test ns, wt = 23, *Atm*^+/−^ = 21, *Atm*^−/−^ = 12; independent experiments = 3) from cultured neurons. **E** E/I ratio from wt, *Atm*^+/−^ and *Atm*^−/−^ cultured neurons show no statistical changes between the three groups (Kruskal–Wallis test followed by Dunn’s multiple comparison test ns, wt = 16, *Atm*^+/−^ = 15, *Atm*^−/−^ = 11; independent experiments = 3).

By recording excitatory and inhibitory postsynaptic currents in miniature (mEPSCs and mIPSCs) we assessed synaptic functions in *Atm*^+/−^ and *Atm*^−/−^ cortical cultures (Fig. 3A, B). Frequency and amplitude of mIPSCs did not display significant variations among the groups (Fig. 3A–C). We detected in *Atm*^+/−^ neurons increases in the amplitude of mEPSCs (Fig. 3B–D) never found in hippocampal preparations (Supplementary Fig. 2B, C), thus suggesting different changes among hippocampal and cortical neurons. Also, the E/I balance resulted similar in the three groups (Fig. 3E). Once again in *Atm*^+/−^ and *Atm*^−/−^ hippocampal cultures, we detected increased inhibitory transmission respect to the wt cells (Supplementary Fig. 2B). Immunofluorescent analysis of vGAT and vGlut1 density in wt and *Atm*^−/−^ hippocampal neurons confirmed increased inhibitory synaptic puncta (Supplementary Fig. 2D) similarly to what we already described in *Atm*^+/−^ cells [15].

### Reduced ATM leads to increased levels of kainate receptors

**Fig. 4:**
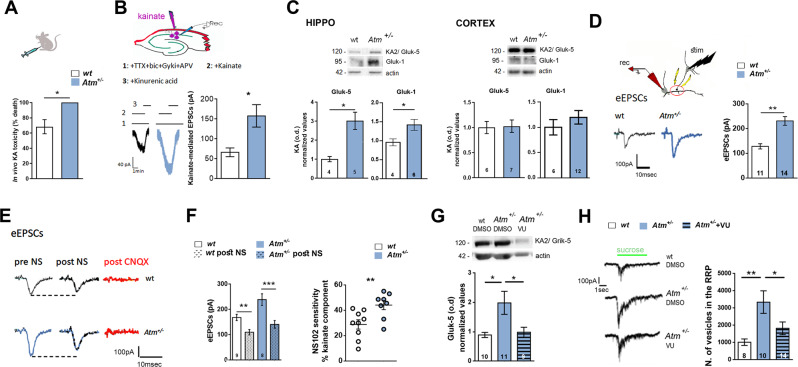
ATM heterozygosity leads to increased levels of functional kainate receptors which are responsible for higher glutamatergic response and bigger size of RRP. **A** Kainate-induced toxicity in male wt (*n* = 13) vs. *Atm*^+/−^ (*n* = 12) mice; KA dosage: 25 mg/kg. *t* test *p* = 0.027. Independent experiments = 3. **B** On top: scheme of the experimental setting. Under the scheme: representative traces (left) and quantification (right) of the kainate-evoked post-synaptic currents in wt and *Atm*^+/−^ mice CA1 pyramidal neurons are showed (EPSCs amplitude: Unpaired *t*-test, *p* = 0.032; wt = 7 cells and *Atm*^+/−^ = 13 cells). Independent experiments = 3. **C** Western blotting analysis of Gluk-1 and Gluk-5 subunits in hippocampal and cortical tissues from wt and *Atm*^+/−^ P40 mice indicating increased protein levels only in the hippocampus of *Atm*^+/−^ animals (Hippo: Gluk-5 Mann–Whitney test: *p* = 0.016; Gluk-1 Mann–Whitney test: *p* = 0.11. Gluk-1 Mann–Whitney test: *p* = 0.65). **D** Picture represents mono-synaptically connected neurons and traces are representative of evoked excitatory postsynaptic currents (eEPSCs) generated by applying 100-mV depolarization pulse in the presynaptic cell. Right: Analysis of eEPSCs displays a significant increase in evoked events induced in *Atm*^+/−^ cultures (eEPSCs: Mann–Whitney test: *p* = 0.0062. Independent experiments = 4. **E** Representative traces of eEPSCs before (pre) and after (post) delivery of KARs antagonist, NS-102. CNQX delivery confirms the glutamatergic origin of eEPSCs. **F** Left panel: Quantification of mean eEPSCs indicates that NS-102 delivery in wt cultures induces a significant reduction in amplitude of evoked currents. A much higher reduction is achieved by NS-102 treatment in *Atm*^+/−^ cultures (eEPSCs: Kruskal–Wallis followed by Dunn’s multiple comparison test: *p* < 0.0001). Right panel: The graphs show the percentages of NS-102-induced reduction of eEPSCs amplitudes (% of reduction: Unpaired *t*-test: *p* = 0.01). Independent experiments = 3. **G** Top: Representative blotting experiment shows that in *Atm*^+/−^ cultures treated with VU at 6-8-10-12 div levels of Gluk-5 subunits are comparable to those found in wt-treated DMSO cultures. Mann–Whitney test: *p* = 0.0068. Independent experiments = 3. **H** Representative traces of sucrose-evoked responses from wt-DMSO, *Atm*^+/−^-DMSO and *Atm*^+/−^-VU neurons and quantitative analysis of number of vesicles included in the readily releasable pool (RRP). Number of vesicles contained in the RRP has been determined by dividing the RRP charge by the mean mEPSC charge for each neuron. Green line indicates the time of sucrose application. Tukey‘s multiple comparison test: *p* = 0.0086. Independent experiments = 2.

Then, we assessed in vivo effects evoked by a single intraperitoneal injection of the convulsant agent kainic acid (KA). We treated only *Atm*^+/−^ animals to exclude unspecific modifications and possible higher susceptibility linked to ATM complete loss. KA treatment generates a toxic effect as indicated by the higher percentage of lethality reached in *Atm*^+/−^ mice respect to the wt group (Fig. 4A). In *Atm*^+/−^ hippocampal slices we found increased excitatory postsynaptic (EPS) currents respect to the wt upon KA delivery (10 µM, Fig. 4B) and by western blotting analysis in total hippocampal homogenates a significant increment of Gluk-1 and Gluk-5 subunits of KA receptors (KARs, Fig. 4C). Once again, differences in KARs signal occurred only in hippocampal but not in cortical lysates collected from *Atm*^+/−^ mice (Fig. 4C). In the attempt to deepen functional consequences linked to the higher KARs levels in *Atm*^+/−^ neurons, we selected mono-synaptically connected neurons and induced evoked unitary postsynaptic currents (eEPSCs). *Atm*^+/−^ hippocampal neurons displayed a wider eEPSC respect to the wt counterpart (Fig. 4D). Thus, we decide to locally apply the kainate antagonist NS-102 (3 µM, Fig. 4E), which is able to reversibly block the peak current induced by KARs activation in cultures [25, 26]. In wt neurons, eEPSCs were reduced to approximately 20% upon NS-102 delivery, whereas in *Atm*^+/−^ cells, eEPSCs were more strongly inhibited by the same treatment (about 40%, Fig. 4F). These results indicate that ATM deficiency affects synaptic composition leading to higher levels of functional KARs whose activation is responsible for increased evoked glutamatergic currents (Fig. 4D–F).

In literature, a functional relation between KARs (Gluk-1 and GluK-2 subunit of KARs) and KCC2 has been demonstrated [27] and in the absence of GluK-2 expression, KCC2 function is reduced [27]. Thus, we asked if in ATM-deficient condition (where we have increased KCC2 levels), the blockade of KCC2 function might conversely restore the increased expression of KARs. To this issue, we treated *Atm*^+/−^ neurons at 6-8-10-12 div with the KCC2 blocker VU0240551 (1 µM) or vehicle [28] and measured by western blotting experiments KARs abundance in mature cultures (Supplementary Fig. 3A). *Atm*^+/−^cultures treated with VU display levels of Gluk-5 subunits significantly reduced compared with those found in DMSO-treated *Atm*^+/−^ cultures (Fig. 4G; no changes in Gluk-1 signals). Then, to further investigate functional consequences of KARs accumulation, we assessed changes in the readily releasable pool (RRP) of synaptic vesicles [29], since presynaptic KARs may change neurotransmitter release promoting clustering of synaptic vesicles in the RRP [30, 31]. To this end, we exploited the puff application (4 s lasting) of hypertonic sucrose solution [32, 33] and found a higher number of vesicles in the RRP in Atm^+/−^ cultures respect to wt cells, nicely normalized by VU delivery during development (Fig. 4H). No changes in the mEPSCs charge were detected among the three groups (Supplementary Fig. 3B). These results indicate that increased levels of KARs (i) accumulate at the presynapse and (ii) generate a larger RRP size. Importantly, both these mechanisms are KCC2-dependent.

### In ATM-deficient condition pre-synaptic KARs do not account for the higher glutamate release probability

**Fig. 5:**
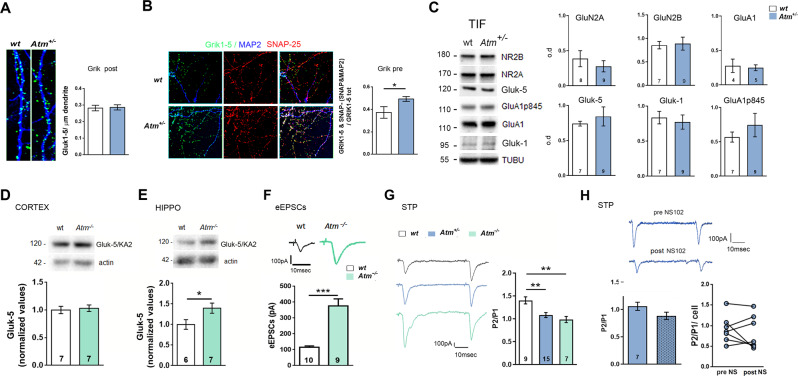
In ATM deficient condition the increased kainate receptors localize at the pre-synapse but do not participate to glutamate release probability. **A** Immunocytochemical experiments for Grik1–5 detection (green) at the postsynaptic compartment in wt and *Atm*^+/−^ cultured neurons per unit length of dendrite (blue; MAP-2 positive filament). Number of cultures: wt = 3, *Atm*^+/−^ = 3; Number of fields per each glass are at least 10. Dendrite length = at least 20 mμ. Mann–Whitney test: *p* = 0.64. **B** Immunocytochemical experiments performed in wt and *Atm*^+/−^ cultured neurons against Grik1–5 (green), MAP-2 (blue), and SNAP-25 (red) to evaluate amount of presynaptic Grik1–5. Since SNAP-25 is also postsynaptic, we excluded Grik1–5 and SNAP25 colocalizing puncta onto MAP-2-positive filaments. Unpaired *t*-test: *p* = 0.017. **C** Left: Representative Triton-insoluble fraction (TIF) lanes. Right**:** TIF analysis on hippocampal tissues from wt and *Atm*^+/−^ mice show comparable levels of Gluk-5 and Gluk-1 KARs subunits (Gluk-5: 2-tailed unpaired *t*-test *p* = 0.55, Gluk-1: 2-tailed unpaired *t*-test *p* = 0.65), no changes in GluA1 or its 845-residue phosphorylated form (GluA1: Mann–Whitney test *p* > 0.99; GluA1-p845: 2-tailed unpaired *t*-test *p* = 0.409) as well as NMDA receptors subunits, GluN2B and GluN2A (GluN2A: 2-tailed unpaired *t*-test *p* = 0.14; GluN2B 2-tailed unpaired *t*-test *p* = 0.84). **D** and **E** Gluk-5 protein levels are increased in the hippocampus of *Atm*^−/−^ mice but not in the cortex. *t* test: *p* = 0.041. **F** Representative traces of eEPSCs in wt and Atm^−/−^ cultures and quantitative analysis (eEPSCs amplitude: Mann–Whitney test: *p* < 0.0001). Independent experiments = 3. **G** Traces of short-term plasticity (STP) experiments. In mono-synaptically connected neurons, we stimulated pre-synapses with two consecutive stimuli at 50 ms of inter-spike interval and quantified PPR (P2/P1). P2/P1: Kruskal–Wallis followed by Dunn’s multiple comparison test: *p* = 0.0003. Independent experiments = 3. **H** Representative traces of short-term plasticity experiments performed in *Atm*^+/−^ neurons before and after NS-102 delivery. Mean analysis of P2/P1 indicate that KARs receptor are not involved in STP mechanisms (P2/P1: Mann–Whitney test: *p* = 0.15). Similar results may be evidenced by the analysis of P2/P1 in each cell (dot) before and after NS-102 delivery (P2/P1: Wilcoxon matched-pairs signed rank test: *p* = 0.34). Independent experiments = 3.

Sucrose experiments suggest that in *Atm*^+/−^ cultures KARs accumulate at the presynapse. However, we produced confocal analysis to further elucidate the pre- and post-synaptic Gluk1-5 localization. We stained hippocampal cultures with antibodies for Gluk1–5 subunits, MAP2 to identify somato-dendritic processes and SNAP-25 as membrane marker. We analysed density of Grik1–5 puncta along MAP2-positive branches of wt and *Atm*^+/−^ hippocampal cultures to evaluate post-synaptic KARs abundance and found comparable immunostaining between the two groups (Fig. 5A). To measure the pre-synaptic Gluk1–5, we analysed colocalization between Gluk1–5 and SNAP-25-positive puncta but, since SNAP-25 is also post-synaptic [34], we excluded in this analysis the SNAP-25 signal along MAP2-positive dendrites. Results in Fig. 5B confirmed the increased KARs signal at the presynaptic compartment.

Finally, we took advantage of the Triton-insoluble fraction (TIF) that is enriched with postsynaptic proteins ([35], Supplementary Fig. 3C). TIF analysis carried out in hippocampi explanted from *Atm*^+/−^ mice showed comparable levels of Gluk-5 and Gluk-1 KARs subunits respect to the wt (Fig. 5C). TIF preparations did not reveal any changes in signals related to AMPA and NMDA post-synaptic receptors (Fig. 5C) as indicated by normal levels of (i) GluA1 or its 845-residue phosphorylated form (GluA1-S845P [35, 36]) and (ii) GluN2B and GluN2A related signal (Fig. 5C). These results confirm that KARs increments are more likely presynaptic. Also, analysis of KARs expression in *Atm*^−/−^ mice revealed increased KARs-related signal only in hippocampal but not in cortical *Atm*^−/−^ homogenates (Fig. 5D, E) and, once again, a higher eEPSCs in *Atm*^−/−^ hippocampal neurons (Fig. 5F).

Presynaptic KARs have been described also as auto-receptors able to regulate glutamate release [37, 38] and probability of release [37, 39]. Thus, we carried out short-term plasticity (STP) experiments [33, 40] since they directly unveil the contribution of presynaptic components in the neurotransmitter release process. We applied in wt, *Atm*^+/−^ and *Atm*^−/−^ hippocampal cultures electrophysiological protocol for STP and evaluated the paired pulse ratio (PPR). *Atm*^+/−^ and *Atm*^−/−^ neurons display reduced PPR respect to wt cells indicating an increased release probability (Fig. 5G). To evaluate KARs contribution in PPR alterations we delivered NS-102 to *Atm*^+/−^ hippocampal neurons but no effects were obtained in terms of A2/A1 mean value (Fig. 5H) suggesting that STP deficits reflect more likely calcium-dependent alterations and not exclusively the aberrant activation of presynaptic KARs. Interestingly, calcium imaging experiments performed in 14 div *Atm*^*+/+*^, *Atm*^+/−^ and *Atm*^−/−^ hippocampal neurons indicate reduced basal calcium levels and calcium transients upon KCl-induced depolarization in *Atm*^−/−^ cultures (Supplementary Fig. 3D). These results indicate impairments in intracellular calcium homoeostasis and VOCCs levels in mature cells.

We then analysed evoked inhibitory postsynaptic currents (eIPSCs) in *Atm*^*+/+*^, *Atm*^+/−^, and *Atm*^−/−^ cultures and found higher responses in *Atm*^−/−^neurons respect to wt preparations (Supplementary Fig. 4A). Application of STP protocol in wt presynaptic GABAergic neurons generates a PPR < 1. Analysis of PPR in *Atm*^+/−^ and *Atm*^−/−^ cultures displayed lower values (Supplementay Fig. 4B) indicating a still higher release probability at GABAergic synapses. These results may be ascribed also to the increased number of GABA-containing vesicles as previously described in *Atm*^+/−^ neurons [12, 15].

### The acute pharmacological inhibition of ATM in wt young adult mice produces both shared and distinct transcriptional signature compared to the ATM genetic model

**Fig. 6:**
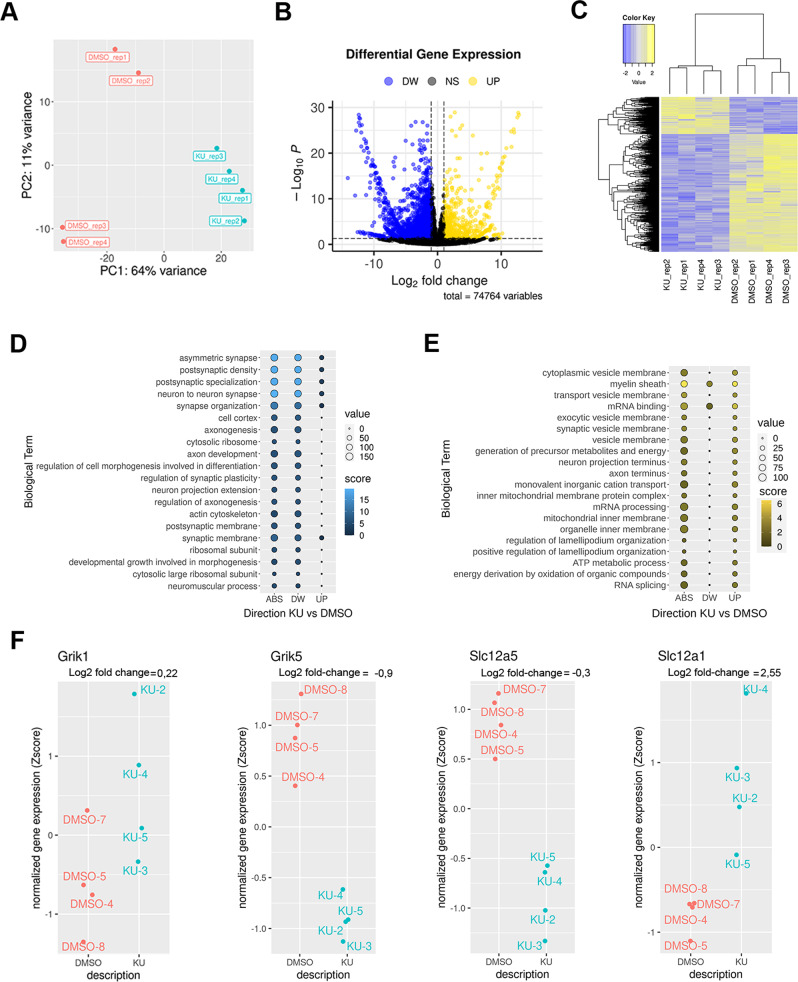
RNA sequencing analysis in hippocampi of wt mice treated with KU55933. **A** Principal-component analysis (PCA) of the gene expression counts (log2 vst-method normalized counts) showing the first versus the second PC. In the axes, the percentage of the explained variance by the component is reported. Samples in the different conditions are highlighted in different colours. **B** Volcano plot highlighting significant differentially expressed genes in the hippocampus of KU55933 vs. vehicle- treated mice. Significantly up-regulated genes shown in yellow (*p* < 0.05, fold change ≥ 2, significantly down-regulated genes shown in blue (*p* < 0.05, fold change ≤ 2); black dots represent non-differentially expressed genes. **C** Heatmap showing the differentially expressed genes (log2 fold change < −1 or >1, multiple tests correction, adjusted *p* < 0.05) in the comparisons. Gene expression counts were normalized by the vst method, hierarchically clustered with average linkage, and Euclidean distance metric was applied. Two main clusters of genes were obtained: the first comprises the genes up-regulated through the samples cluster and a second one comprises the genes down-regulated. **D**–**F** Functional annotation: bubble plots graphs represent the enrichment of the biological terms based on the combined score (*x*-axis), as calculated by the clusterProfiler; in each column, the dots represent the Biological Terms enriched in the functional analyses of the differential genes in both directions (ABS), in up-regulated (UP) genes or in down-regulated (DW) genes in KU vs. DMSO comparison. Since this tool provides a large collection of elements, the top elements (*n* = 15) in elements were selected for down -regulated (left panel) genes or for the UP-regulated (right). All of the terms have multiple test correction adjusted *p*-value < 0.05. The size of each dot is proportional to the number of the genes enriched in the term and the colour is proportional to the—log 10 *p* value (score).

To directly verify that ATM-dependent synaptic modifications occur during neuronal development, we treated young adult wt mice with a single intranasal injection of the selective ATM kinase inhibitor KU55933 (KU) and performed RNA sequencing (RNA seq) analysis in the hippocampal tissues explanted 2 days later. Principal component analysis (PCA) exhibits a pattern of gene expression in KU mice that was clearly distinct from those of the vehicle-treated mice (Fig. 6A). Unsupervised hierarchical clustering analysis revealed a clear separation between the KU- and vehicle-treated hippocampal samples and similarities amongst the replicates (Fig. 6C). The distribution of differentially expressed transcripts between KU and vehicle treated mice were plotted in a volcano plot (Fig. 6B). A total of 766 and 2432 transcripts were up- and down-regulated, respectively (Fig. 6B, C). Supplementary Table 1 shows the entire list of hippocampal transcripts differentially expressed. We functionally categorize the differentially expressed genes (DEGs) by Gene Ontology (GO) analysis and found that the up-regulated genes and downregulated genes belong to distinct functional categories. Among the most represented functional categories (GO: BP, MF, and CC) in the down-regulated genes several terms were related to postsynaptic density composition and specialization, synaptic organization and plasticity (Fig. 6D). Conversely, among the up-regulated genes we discovered a significant enrichment in genes related to presynaptic elements such as cytoplasmic vesicles membrane and transport vesicles membrane (Fig. 6E). In accordance with the role of ATM in metabolic pathways, we find a significant over-representation of terms related to mitochondrial regulation (Fig. 6E). Focusing the analyses to the gene level of *Grik-1, Grik-5, Scl12a5*, and *Scl12a1* we noted that *Grik-1* displayed only a marginal increment, whereas *Grik-5* was significantly reduced. Also, *Slc12a1*, i.e. the gene which codifies for NKCC1 cotransporter, does not change and *Slc12a5* which codifies for KCC2, is significantly reduced (Fig. 6F). Thus, our bioinformatics results do not indicate a clear or strong enhancement in terms of kainate receptors upon the acute inhibition of ATM kinase activity in wt adults thus confirming that synaptic changes here identified are more likely reflecting developmental changes. Accordingly, no upregulation in *Slc12a5* transcription has been reported corroborating the link between KCC2-KARs. Finally, among deregulated genes, we also found variations in the calcium channels genes (*Cacna1, Cacnb*) and Cac*Slc24a4* gene which encodes the NCKX4, a ion exchanger that extrudes calcium across the plasma membrane [41].

We next analysed the hippocampal DEGs of KU55933 treated mice to verify whether they were enriched for genes that have been previously associated to A-T or ATM, using the Enrichr analysis tool (“NURSA Human Endogenous Complexome” and “Gene perturbations” set libraries, respectively). We found a significant enrichment for ATM-interacting partners “ATM (Ab-3) (ATM)” (adjusted *p* = 0.02) and for genes deregulated in cellular systems stably knocked-down for ATM “ATM knockdown human GSE1676 sample 1865” (adjusted *p* = 0.01). We also tested our DEGs for enrichments in genes differentially expressed in hiPSC-derived neurons from fibroblasts of an A-T patient and in ATM knockout hNPCs, both gene sets compiled from an RNA sequencing study [42]. Overall, we observed a significant enrichment for both genes sets (*p* < 0.05). These results reveal partial transcriptional similarities between the genetic condition and pharmacological inactivation of ATM in adulthood but a complete difference regarding our genes of interest.

To further demonstrate that acute ATM inhibition in adults is not responsible for KARs increments, we challenged the valproic acid (VPA) mouse model for autism spectrum disorder, a model of autism where we described increased ATM expression. Here, ATM inhibition by intranasal KU, rescued autistic-like defects [28]. KARs expression in hippocampi explanted from adult controls and in VPA animals treated once with KU or DMSO [28] did not reveal any differences among the groups (Supplementary Fig. 5A). Also, in hippocampal tissues explanted from *Atm*^+/−^ mice prenatally treated with Fluox we did not rescue KARs expression subunits (Supplementary Fig. 5B) as expected, since by Fluox we restored NKCC1 expression leaving unchanged KCC2.

In conclusion, our results show that reduced ATM levels in the hippocampus control development and composition of excitatory and inhibitory synapses by a KCC2-dependent mechanism.

## Discussion

In this study we display how ATM shapes development of glutamatergic synapses. By exploiting *Atm*^+/−^ and *Atm*^−/−^ mice we unveiled the ATM context-specificity in terms of excitatory synapses composition, GABAergic development and KCC2 changes. Here, we demonstrate that the increased amount of KCC2 leads to the accumulation of presynaptic KARs at excitatory synapses. This correlates with enhanced clustering of synaptic vesicles which contributes to stronger glutamatergic current upon evoked stimulation. Inhibition of KCC2 activity normalizes the increased kainate receptors levels and also number of presynaptic vesicles. We cannot exclude that the increased kainate receptors expression strengthens GABAergic inhibition via a metabotropic and ionotropic mechanism [43]. In this view, our data confirm the importance of the KARs/KCC2 functional link in excitatory/inhibitory balance in the hippocampus [43] and now we indicate a new mechanism mediated by ATM activity. Also, ATM deficiency affects calcium-dependent pathways as evidenced by results collected in (i) the genetic model (calcium imaging data in mature cells) and (ii) RNA seq investigation performed in hippocampi of mice treated with the ATM inhibitor KU. In RNA-sequencing experiments we find downregulation of genes encoding for subunits of VOCCs, (*Cacna1* and *Cacnb* genes) and changes of gene codifying for potassium-dependent sodium calcium exchanger (*Slc24a4* gene), designated NCKX4 [41] also described in iPSCs differentiated neurons established from fibroblasts of Ataxia Telangiectasia (A-T) patients [42]. These results may suggest a rational explanation for reduced calcium transients and changes in short-term plasticity in mature ATM deficient neurons.

KARs dysregulation may be at the basis of the drug-resistant seizures and developmental delay described in subjects with genetic mutations linked to other DNA repair proteins as in Breast Cancer 1-associated Ataxia Telangiectasia mutated activation-1 protein (BRAT1) gene [44–46]. Also, four mutations in the codifying gene for the polynucleotide kinase/phosphatase (PNKP) have been described and linked to early-onset intractable seizures and developmental delay (MCSZ). PNKP operates in the majority of DNA breaks [47] upon phosphorylation from ATM [48].

Finally, this study shows the unexpected ability of Fluoxetine to promote NKCC1 levels. The choice to follow a “prenatal Fluoxetine” protocol is based on two purposes. Our first intent was to demonstrate that cognitive phenotype linked to A-T condition is reversible by an early approach. Since KCC2 is mainly expressed postnatally in mouse brain, we excluded the possibility to balance KCC2 levels by a prenatal treatment. Indeed, we hypothesised to act on GABAergic system, as Fluoxetine is known to affect GABA transmission [49]. Surprisingly, we show increased levels of the chloride co-transporter NKCC1. Thus, even if our results suggest positive outcomes of Fluoxetine treatment, its prenatal delivery to humans must be approached with caution. On the contrary, the chronic Fluoxetine treatment in ATM adult mice would be easily translated to patients. Based on our results, Fluoxetine corrects spatial deficits in *Atm*^−/−^ mice, leaving unchanged the non-spatial learning impairments. Actually, the fact that Fluoxetine normalizes specific behavioural defects is not surprising as already described in patients with vascular cognitive impairments, where Fluoxetine ameliorates some executive functions leaving unchanged other cognitive domains [50].

## Experimental procedures

### Animals

All the experimental procedures followed the guidelines established by the Italian Council on Animal Care and were approved by the Italian Ministry of Health (authorization nos. 991/2016-PR and 369/2019-PR). All efforts were made to minimize the number of animals used and their sufferings. Mice were maintained under standard laboratory conditions [room temperature (22 ± 2 °C) with 12:12 h light:dark cycle (lights on at 8.00 AM) with food and water ad libitum]. Mice were kept maximum 5 per cage. Tests were conducted during the light phase of the circadian cycle between 9.00 AM and 1.00 PM.

### Genotyping

Genotyping for ATM animals was performed using polymerase chain reaction (PCR) technique. After DNA purification [51], 3 μl of DNA were added to: 7.5 μl of master mix (GoTaq, Promega), 0.25 μl of each primer (Sigma-Aldrich) and 3.75 μl of Nuclease free water (Promega). The DNA was amplified using a thermocycler (Biorad).

Primers sequences for *Atm* genotyping: 5′-GTAGTAACTATTAGTTTCGTGCA-3′, 5′-TAGGGTGTAGTAGTGGAGGA-3′, 5′-ACGTAAACTCGTCTTCAGACCT-3′.

### Behavioural tests

Spontaneous alternation (SA) test: Spontaneous alternation was measured using a Y-shaped maze constructed with three symmetrical grey solid plastic arms at a 120° angle (26 cm length, 10 cm width, and 15 cm height) as previously described [52]. Male mice were individually placed in the centre of the maze and were allowed to freely explore the three arms for 8 min. Arm entry was defined as all four limbs within the arm. A triad was defined as a set of three arm entries, when each entry was in a different arm of the maze. The maze was cleaned with water and 70% ethanol between sessions to eliminate odour traces. The number of arm entries and the number of triads were recorded in order to calculate the alternation percentage (generated by dividing the number of triads by the number of possible alternations and then multiplying by 100). The number of animals used are specifically indicated in the graph.

### In vivo Fluoxetine treatment

Three to six months old *Atm*^+/−^ females were mated for 24 h with an *Atm*^+/−^ male. Starting from gestational day 0 (GD0) females were weighed every 2 days till GD10. At GD10 females who gained at least 2 g, gradually in time, were treated subcutaneously with 10 mg/kg body weight Fluoxetine or vehicle every day from GD10 to delivery (GD19). Fluoxetine solution was prepared fresh every day. For fluoxetine “adult protocol” *Atm*^−/−^ adult males were treated for 3 weeks every day with a subcutaneous fluoxetine injection (10 mg/kg body weight).

### TIF preparation

Triton insoluble fraction (TIF) is a fraction highly enriched in postsynaptic density proteins (i.e. receptor, signalling, scaffolding and cytoskeletal elements) absent of presynaptic markers [53, 54]. Mouse hippocampi were homogenized at 4 °C in a the ice-cold buffer with Roche cOmplete^TM^ Protease Inhibitor Cocktail, Ser/Thr and Tyr phosphatase inhibitors (Sigma-Aldrich), 0.32 M Sucrose, 1 mM Hepes, 1 mM NaF, 0.1 mM PMSF, 1 mM MgCl_2_ using a glass-teflon homogenizer. An aliquot of total homogenate was kept for Western Blot analysis.

Homogenate samples were centrifuged at 13,000 × *g* for 15 min at 4 °C. Triton-X extraction of the resulting pellet was carried out at 4 °C for 15 min in an extraction buffer (0.5% Triton-X, 75 mM KCl and protease inhibitors (Roche cOmpleteTM Protease Inhibitor Cocktail). After extraction, the samples were centrifuged at 100,000×*g* for 1 h at 4 °C and the TIFs obtained were resuspended in 20 mM HEPES with protease inhibitors (Roche cOmpleteTM Protease Inhibitor Cocktail).

### Western blotting

Proteins were extracted starting from explanted hippocampal and cortical tissues from P40 wt, *Atm*^+/−^ and *Atm*^−/−^ animals. Lysis buffer contained 1% sodium dodecyl sulphate (SDS), 62.5 mM Tris–HCl (pH 6.8), 290 mM sucrose and protease inhibitors (Roche cOmpleteTM Protease Inhibitor Cocktail). Protein concentration was estimated using Bicinchoninic Acid Assay (BCA) kit (Thermo Fisher Scientific) using a bovine serum albumin-based standard curve. For Western blot analysis, equal amounts of proteins (20 μg) were loaded and run on 10% or 12% acrylamide gel and separated by SDS–PAGE electrophoresis. Nitrocellulose membranes were incubated with the following primary antibodies: rabbit anti-KCC2 1:1000 (Millipore), rabbit anti-Mecp2 1:1000 (Sigma-Aldrich), rabbit anti-NKCC1 1:500 (Millipore), mouse anti-PSD95 1:1000 (NeuroMab), rabbit anti Gluk-5/KA2 1:1000 (Millipore), mouse anti-SNAP25 1:1000 (Chemicon), rabbit anti-vGlut1 1:1000 (Synaptic Systems), rabbit anti-vGAT 1:1000 (Synaptic Systems), rabbit anti-GluN2A 1:1000 (Invitrogen), mouse anti-GluN2B 1:1000 (Neuromab), mouse anti-GluA1 (Cell signalling), rabbit anti-GluA1-S845p (Millipore), mouse anti-tubulin 1:10,000 (Sigma-Aldrich), anti-Grik1 1:500 (Sigma- Aldrich). Mouse anti-actin 1:1000 (Sigma-Aldrich) was used as loading control.

HRP-conjugated secondary antibodies (Jackson ImmunoResearch) were used 1:40,000.

Immunoreactive bands were detected by using the Pierce ECL Western Blotting Substrate (Thermo Fisher Scientific), acquired with Chemidoc and analysed using ImageJ software (NIH, Bethesda, MD, USA).

### Brain slice electrophysiology

Coronal hippocampal slices (thickness, 250–400 μm) from C57Bl/6 Atm^+/+^ and Atm^+/−^ 30 days old male mice (P30) were prepared as previously described [55]. Slices containing the hippocampus were transferred to a recording chamber and perfused with artificial CSF (aCSF, containing in mM: 125 NaCl, 2.5 KCl, 1.25 NaH_2_PO_4_, 1 MgCl_2_, 2 CaCl_2_, 25 glucose, and 26 mM NaHCO_3_ (pH 7.3)) at a rate of ~2 mL/min and at room temperature. Whole-cell patch-clamp electrophysiological recordings were performed with a Multiclamp 700B amplifier (Axon CNS Molecular Devices, USA) and using an infrared-differential interference contrast microscope (Nikon Eclipse FN1). Patch electrodes (borosilicate capillaries with a filament and an outer diameter of 1.5 μm; Sutter Instruments) were prepared with a four-step horizontal puller (Sutter Instruments) and had a resistance of 3–5 MΩ. CA1 pyramidal neurons post-synaptic currents were recorded using a caesium chloride-based intracellular solution (in mM: 140 CsCl, 2 MgCl_2_, 1 CaCl_2_, 10 EGTA, 10 HEPES–CsOH, 2 ATP (disodium salt) (pH 7.3 with CsOH). Neurons were whole-cell patch clamped to −65 mV and kainate receptor-mediated post-synaptic currents were recorded in aCSF supplemented with kainate (10 µM). Throughout the experiments, in order to isolate kainate receptor-mediated currents, aCSF were supplemented with GYKI53655 (10 µM, Sigma Aldrich), APV (100 µM, Sigma Aldrich) and bicuculline (20 µM, Sigma Aldrich) to block AMPA, NMDA and GABAA receptors respectively. Additionally, tetrodotoxin (3 µM, Tocris) were used to inhibit calcium-dependent release of glutamate from the pre-synapse. Kainate receptors-mediated currents were blocked using kynurenic acid (3 mM, Sigma-Aldrich), a broad-spectrum blocker of glutamatergic transmission.

### In vivo Kainic Acid treatment

Kainic Acid (KA, Sigma-Aldrich) was dissolved in saline solution and administered intraperitoneally 25 mg/kg body weight. Adult (P90) wt and *Atm*^+/−^ male animals were used. Seizure severity was determined according to the Racine’s scale: stage 0: normal behaviour; stage 1: immobility; stage 2: forelimb and/or tail extension, rigid posture; stage 3: repetitive movements, head bobbing; stage 4: forelimb clonus with rearing and falling (limbic motor seizure); stage 5: continuous rearing and falling; stage 6: severe whole body convulsions (tonic-clonic seizures); stage 7: death. For each animal, the rating scale value was scored every ten minutes for a maximum of 3 h after KA administration. Data collected were used to calculate the KA toxicity (% of death) for each experimental group. We excluded mice that died within 20 min following KA injection (*n* = 1) since it is indicative of intravenous rather than intraperitoneal kainate delivery. Also, we excluded 3 animals (1 wt + 2 ATM het) since they did not experience any hyperexcitability behaviour after kainate injection. These criteria were pre-established.

### Cell cultures

Hippocampal and cortical neurons were established from E18 mice littermates as previously described [56]. Briefly, tissues were isolated from the brain and, after trypsinization, were dissociated and plated onto glass coverslips previously coated with poly-l-lysin (Sigma-Aldrich). Cultures were grown in Neuronal medium containing B-27 supplement (Gibco) and GlutamaX (Invitrogen) at 37 °C and 5% CO_2_.

### Calcium imaging

DIV 5 (or DIV 14 for experiments in mature cultures) hippocampal and cortical neurons were loaded with the membrane-permeable fluorescent Ca^2+^ indicator Fura2-AM (1 μM; Sigma-Aldrich) for 30 min at 37 °C, 5% CO_2_. Cells were then washed with KRH buffer (NaCl 125 mM, KCl mM, MgSO_4_ 1.2 mM, KH_2_PO_4_ 1.2 mM, CaCl_2_ 2 mM, Hepes 25 mM, d-glucose 6 mM) and placed into the recording chamber of an inverted microscope (Axiovert 100, Zeiss) equipped with a calcium imaging unit and imaged through a ×40 objective (Zeiss). Fura-2AM was excited at 380 nm and at 340 nm through a Polychrom V (TILL Photonics GmbH) controlled by the TillVisION software 4.01. Emitted light was acquired at 505 nm at 1 Hz, and images collected with a CCD Imago-QE camera (TILL Photonics GmbH). Calcium transients have been addressed by evaluating the fluorescence ratio F340/380. This parameter was recorded regions of interest (ROIs) corresponding to neuronal cell bodies and analysed along sequential images to follow temporal changes. Basically, after a period of basal recording, GABA was administered at 100 μM concentration and increments in F340/380 ratio (ΔF340/380), which represents calcium transient), were considered if higher than 0.05 units. Transients occurring within 5 s after drug administration were considered actual calcium responses. After GABA administration neurons were washed with KRH and let recover for few minutes, then KCl 50 mM was administered to identify viable neurons. Neurons responding to depolarizing stimulus (KCL) with a ΔF340/380 smaller than 0.1 units were excluded from the analysis. We let neurons recover for 20 min after KCl stimulation and repeated this protocol on other two different ROIs in the coverslip.

### In vitro electrophysiology

Whole-cell patch-clamp recordings of EPSCs and IPSCs were obtained from 13–14-day-old hippocampal or cortical neurons using a Multiclamp 700 A amplifier (Molecular Devices) and pClamp-10 software (Axon Instruments, Foster City, CA). mEPSCs or mIPSCs were recorded in presence of tetrodotoxin (1 mM). Evoked currents were recorded in isolated pairs of neurons in low-density cultures. Neurons were held at 70 mV, and eEPSC or eIPSC evoked by a 100-mV depolarization pulse in the presynaptic cell lasting 1 ms. At least 5 stable sweeps for each paired recording have been analysed. NS-102 has been acutely added at 3 μM concentration (Sigma-Aldrich) to eliminate Kainate-mediated current whereas AMPA-dependent currents were removed by 20 μM CNQX (Tocris). In the short-term plasticity experiment, we identified excitatory synapses by electrophysiological kinetics and pharmacological sensibility as in ref. [57] and PPR is the ratio between the amplitude of the second response, A2, respect to the first one, A1 and so PPR = A2/A1. For RRP measurements, hypertonic solution containing 3 M sucrose was infused with a puffer pipette for 4 s. Number of vesicles contained in the RRP of neurons has been determined by dividing the RRP charge by the mean mEPSC charge for each neuron (mEPSCs charge comparable between the two groups).

### Immunocytochemical staining

Immunocytochemical staining was carried out using the following antibodies: rabbit anti-Grik1 and anti-Grik5 (1:500), guinea pig anti-MAP2 (1:500), mouse anti-SNAP25 (1:500), mouse anti-tubulin (1:500), guinea pig anti-vGlut1 (1:1000), rabbit anti-vGAT (1:1000) all of them from Synaptic System. Secondary antibodies were conjugated with Alexa-488, Alexa-555, or Alexa-633 fluorophores (Invitrogen, San Diego, CA, USA). Images were acquired using an Leica SPE confocal microscope with ×63 objective. The number of vGAT and vGlut1-positive puncta has been counted after the detection of an appropriate threshold which was set to 2.5‐fold the level of background fluorescence referring to diffuse fluorescence. For the analysis, vGAT- and vGlut-1-positive puncta per unit length of neuronal processes (tubulin-positive immunoreactivity) have been measured. Regarding vGAT analysis, field per field, isolated processes have been analysed in segments of at least 20 µm and in total, 170 segments have been analysed in wt cultures and 170 in Atm^−/−^ cultured neurons. Regarding vGlut1 analysis, field per field, isolated processes have been analysed in segments of at least 20 µm and in total, 105 segments have been analysed in wt cultures and 114 in Atm^−/−^ cultured neurons. Similarly, we analysed Grik1–5 density along MAP2 positive branches. For this analysis, field per field, isolated dendritic branches have been analysed in segments of at least 20 µm and in total, 135 dendritic segments have been analysed in wt cultures and 163 in het cultured neurons. The number of Grik1–5&SNAP-25 colocalizing puncta was obtained counting the number of positive Grik1–5 puncta present on presynaptic SNAP-25-positive neuronal branches; to specifically identify the presynaptic SNAP-25 fluorescence, the signal corresponding to MAP2-positive branches was subtracted to SNAP-25-positive signal. Fluorescence image processing and analyses were performed with ImageJ Software (National Institutes of Health).

### Intranasal KU55933 treatment in wt mice

KU55933 or vehicle (DMSO) was administered to P40 wt mice by intranasal route at a dosage of 7.5 mg/kg. The mice had been previously anesthetized, and the total volume was administered 3 μL at a time, alternating the 2 nostrils. Hippocampal tissues have explanted 48 h later for RNA extraction.

### RNA extraction and library preparation

Hippocampal RNAs from mice intranasally treated with the Atm kinase blocker KU55933 (*n* = 4) or vehicle (4% DMSO in saline; *n* = 4) were purified using standard column purification according to the manufacturer’s protocol (RNAeasy Mini Kit, QIAGEN). All procedures were conducted in RNAase-free conditions. RNA concentration was evaluated using Qubit RNA BR Assay Kit (Life Technologies). RNA purity was assessed by determining UV 260/280 and 260/230 absorbance ratios using a Nanodrop^®^ ND‐1000 spectrophotometer (Thermo Fisher Scientific). RNA quality was evaluated by measuring the RNA integrity number (RIN) using an Agilent RNA 6000 Nano Kit with an Agilent 2100 Bioanalyzer (Agilent Technologies, Santa Clara, CA, USA) according to the manufacturer’s instructions. Only RNA with a RNA integrity number (RIN) of >6.5 and a DV200 values (% of RNA fragments >200 nucleotides) greater than 89% were selected and used for RNA-seq library preparation.

Libraries were prepared with the TruSeq^®^ Stranded mRNA Sample Preparation kit (Illumina, San Diego, CA, USA) according to manufacturer’s instructions. For each sample of hypothalamus and PFC, 1000 and 500 ng were used as input quantity, respectively. The libraries were sequenced at an average read-depth of 58 million reads per sample on an Illumina NovaSeq 6000 (NovaSeq Control Software 1.7.0) with 2 × 150 bp paired-end protocol, using an SP Reagent Kit (300 cycles) in standalone mode, and libraries loaded at 2.25 nM and a volume of 100 μl. The NovaSeq 6000 sequencing was performed by the LaBSSAH/CIBIO NGS Core Facility of the University of Trento.

### RNA-seq analysis

Raw sequence files were subjected to quality control analysis using FastQC (v 1.3) (http://www.bioinformatics.babraham.ac.uk/projects/fastqc/, accessed on May 2021). Transcript quantification was conducted with SALMON (version v1.4) using Transcriptome index for salmon, with selective alignment method that Improves quantification accuracy compared to the regular index downloaded from http://refgenomes.databio.org/ (accessed on May 2021). the generated gene counts were analysed using DESeq2 package. Starting from the normalized (vsd method) expression matrix, we explore the high-dimensional property of the data using principal component analysis (PCA), as dimensionality reduction algorithm implemented in stats DESeq2 package. Genes that were considered as differentially expressed (DEG), were analysed using hierarchical clustering method (eucledian distance). An adjusted *p*-value cut off of 0.05 and a log fold change >1 or <1 were decided as threshold for detection of DEGs. Supplementary Table 1 shows the entire list of hippocampal transcripts differentially expressed in KU-treated mice.

Visualization of the clustering and heatmap of log2-normalized values were obtained using heatmap.2 of gplots package. Transcripts with a fold change ≥ 2-fold and an adjusted *P*-value < 0.05 were considered as statistically significant differentially expressed. Visualization of the DEGs in the volcano plot was obtained using EhnancedVolcano package. Functional annotation was performed using both clusterProfiler and enrichR Bioconductor packages, whose results were visualized with ggplot2 and ggpubr packages, with ggballonplot functions. In particular clusterProfiler was used for Gene Ontology analysis and enrichR for other databases such as NURSA Human Endogenous Complexome and Gene Perturbations.

NURSA Human Endogenous Complexome database contains proteins identified in complexes isolated from cultured cell. Protein complexes were measured by immuno-precipitation followed by mass spectrometry. Gene perturbations is a database of gene signatures that have been extracted and manually curated from the published literature. The GO enriched annotation results are provided in the Supplementary Table 2.

Furthermore, we performed direct enrichment analysis in our differentially expressed genes, using the hypergeometric test present in R (*P*-value cut-off 0.05), for A-T-associated genes from the gene dataset of ref. [42]. For this analysis, we downloaded two different lists of DEGs from hiPSC-derived neurons established from dermal fibroblasts of A-T patients and hiPSC-derived neurons from healthy controls in which the ATM gene was knockout by CRISPR, respectively [42]. To focus the enrichment analysis on hippocampal expressed genes we used as background a list of tissue-specific expressed genes from our RNA-seq data. This background list was obtained by filtering the genes by the normalized expression values and excluding the ones with the lowest expression levels (<10th percentile).

Hippocampal gene expression dataset of KU55933 or DMSO treated mice have been deposited in the NCBI’s Gene Expression Omnibus (GEO) database (accession number will be available after the publication of the manuscript)

### Statistics

The results are presented as mean ± s.e.m. The normal distribution of experimental data was assessed using D’Agostino–Pearson normality test. To compare two normally distributed sample groups, we used Student’s two-tailed unpaired *t*-test. To compare two sample groups that were not normally distributed we used Mann–Whitney’s non-parametric test. To compare more than two normally distributed sample groups, we used one-way ANOVA, followed by Tukey’s multiple comparisons test, or Kruskal–Wallis test followed by Dunn’s multiple comparison if not normally distributed. Statistical analysis was performed by using SigmaPlot (Systat), GraphPad (Prism) or OriginPro (OriginLab) software. Analysis of variance between the groups is evaluated for each experiment. *P* value of < 0.05 was considered statistically significant. All tests used are two-sided. Analysis of the statistical variability and of the smaller number of animals/cells necessary to reach solid and realistic results has been obtained by analysing the statistical power and by previous publications and ethically in accordance to the principles of the 3Rs.

As methods of randomization, animals of each litter were randomly assigned to the different treatment groups with approximately equivalent numbers in each condition. Then, we replicated this protocol at least two times (thus at least starting from two different litters, according to the numerosity).

Similar approach has been followed for in vitro experiments: we randomly assigned a similar number of Petri dishes to each experimental condition and repeat the experiments at least three times, thus starting from at least three different neuronal preparations.

Researchers were blind to the treatment/genotype condition. When they were not blind, the experimental analysis has been conducted in blind.

Regarding the biochemical data, protein signals resulted undetectable due to technical limits have been excluded.

## Supplementary information

Supplementary Figure 1

Supplementary Figure 2

Supplementary Figure 3

Supplementary Figure 4

Supplementary Figure 5

Supplementary Table 1

Supplementary Table 2

Supplementary material (legends and methods)

UNCROPPED original western blots

Reproducibility checklist

## Data Availability

The data analysed during this study are included in this published article and the supplemental data files. Additional supporting data are available from the corresponding authors upon reasonable request.
