# Supplementary figures and images for: ATM rules neurodevelopment and glutamatergic transmission in the hippocampus but not in the cortex
Source: Cell Death Dis. 2022 Jul 16;13(7):616. doi: 10.1038/s41419-022-05038-7 (PMC9288428; doi:10.1038/s41419-022-05038-7)

# Supplementary Figure 1

**A**

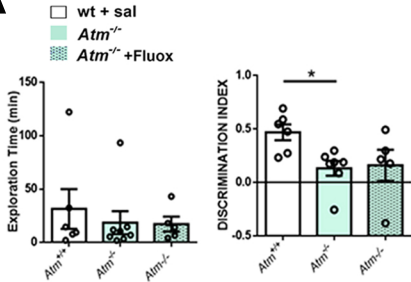

**B**

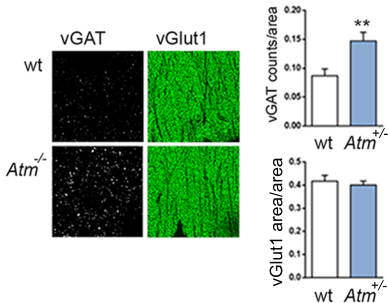

Supplement: Supplementary file 1 — Supplementary Figure 1 [file 41419_2022_5038_MOESM1_ESM.pdf]

# Supplementary Figure 2

**A**

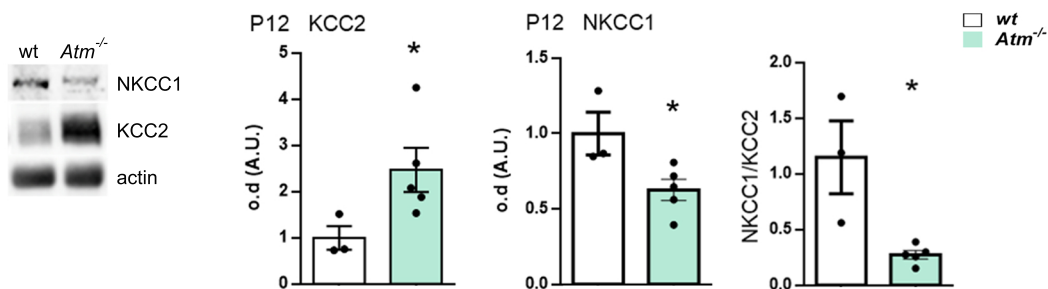

**B**

Ephy in hippo cultures

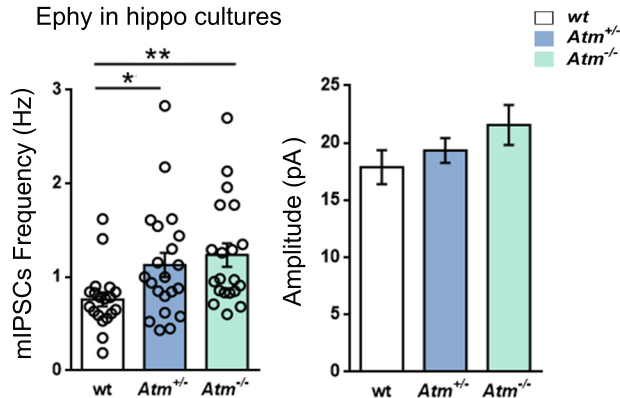

**C**

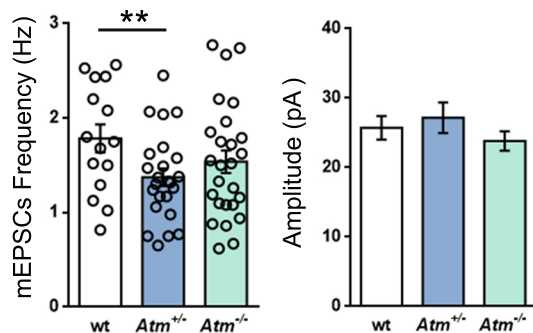

**D**

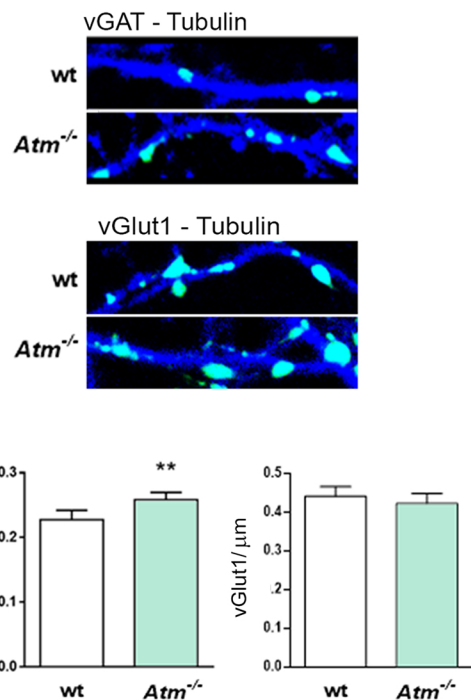

Supplement: Supplementary file 2 — Supplementary Figure 2 [file 41419_2022_5038_MOESM2_ESM.pdf]

# Supplementary Figure 3

**A**

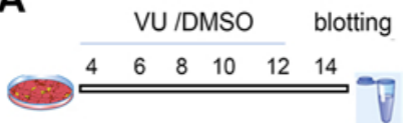

**B**

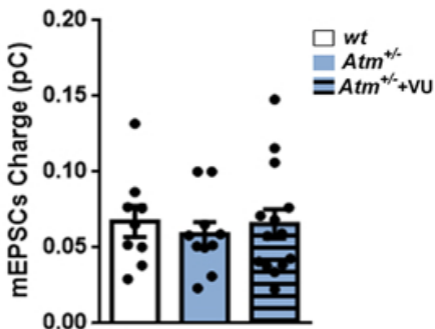

**C**

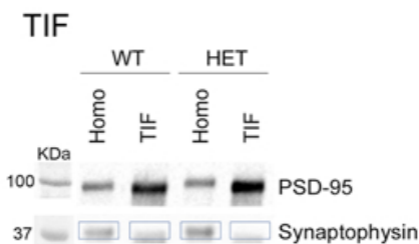

**D**

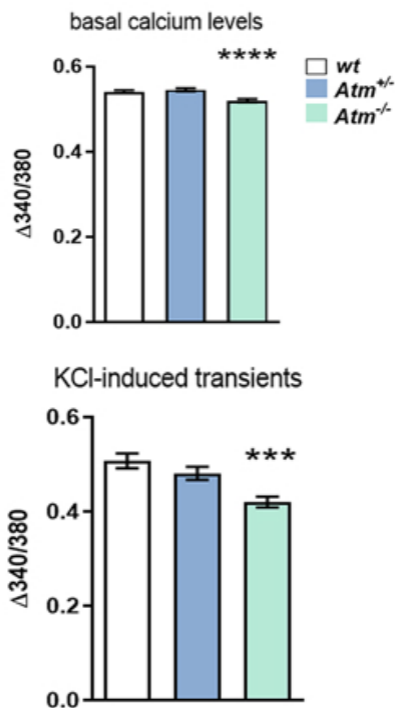

Supplement: Supplementary file 3 — Supplementary Figure 3 [file 41419_2022_5038_MOESM3_ESM.pdf]

# Supplementary Figure 4

## A

eIPSCs

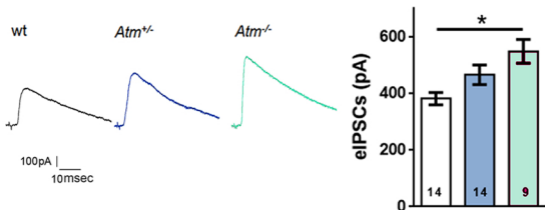

## B

Short term plasticity

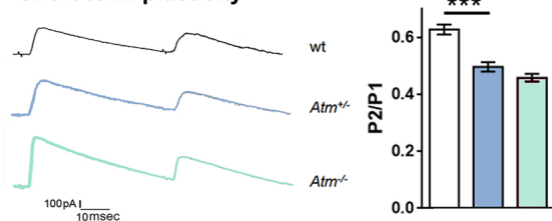

Supplement: Supplementary file 4 — Supplementary Figure 4 [file 41419_2022_5038_MOESM4_ESM.pdf]

# Supplementary Figure 5

**A**

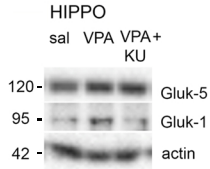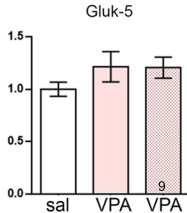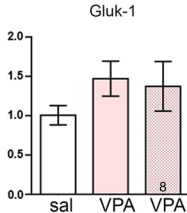

**B**

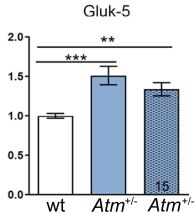

Supplement: Supplementary file 5 — Supplementary Figure 5 [file 41419_2022_5038_MOESM5_ESM.pdf]
