# Supplementary material for: ATM rules neurodevelopment and glutamatergic transmission in the hippocampus but not in the cortex
Source: Cell Death Dis. 2022 Jul 16;13(7):616. doi: 10.1038/s41419-022-05038-7 (PMC9288428; doi:10.1038/s41419-022-05038-7)
Supplement: Supplementary file 8 — Supplementary material (legends and methods) [file 41419_2022_5038_MOESM8_ESM.docx]

**Legends**

**Supplementary Figure 1. (A)** Exploration time (left) and discrimination Index (right) in the Novel Object Recognition (NOR) test in wt, *Atm^-/-^*+ sal and *Atm^-/-^*+ Fluox treatment in adult mice. *Atm^-/-^* mice display lower abilities respect to wt animals that are not rescued by Fluoxetine delivered for three weeks. Kruskal-Wallis test followed by Dunn’s multiple comparison test: p=0,019. Each dot represents a single mouse. **(B)** Immunohistological experiments for vGAT and vGlut1 detection in the stratum radiatum of hippocampus and relative quantification in hippocampi of P40 wt and *Atm^+/-^* mice. VGlut1 and VGAT areas were calculated as the total area of the positive puncta within a selected Region Of Interest (ROI), normalized to the total area of the ROI. Number of animals: wt=2 and *Atm^+/-^*=2.

**Supplementary Figure 2. (A)** Western blotting experiments show NKCC1 and KCC2 levels in P12 wt vs *Atm^-/-^* animals. NKCC1 daat: Unpaired t-test: p=0,036. KCC2 data: Mann Whitney test: p= 0,035. NKCC1/Kcc2 ratio: unpaired t-tets: p=0,011. **(B)** Analysis of frequency and amplitude of inhibitory postsynaptic current in miniature (mIPSCs); frequency: Kruskal-Wallis followed by Dunn’s multiple comparison test: p=0.0036; amplitude: Ordinary One Way Anova followed by Holm-Sidak's multiple comparisons test: p= 0.21). **(C)** Analysis of frequency and amplitude of excitatory postsynaptic current in miniature (mEPSCs); frequency: Ordinary One Way Anova followed by Holm-Sidak's multiple comparisons test: p=0.0074; amplitude: Ordinary One Way Anova followed by Holm-Sidak's multiple comparisons test: p= 0.39). **(D)** Immunocytochemical experiments for vGAT and vGlut1 detection (green) along neuronal processes identified by β-III tubulin labelling in wt and *Atm^-/-^* cultured neurons (blue; β−tubulin-positive filament). Number of cultures: wt=4, *Atm^-/-^*=4. vGAT results: Mann Whitney test: p=0,0013. vGlut1 data: Mann Whitney t-test: p=0,36.

**Supplementary Figure 3. (A)** Scheme indicating cultures treatment during neuronal development with VU0240551 1µM. **(B)** Analysis of mEPSCs charge in wt, *Atm^+/-^* and in *Atm^+/-^* +VU. One Way Anova, followed by Tukey’s multiple comparison test: p=0,82. **(C)** Western blot analysis of post-synaptic and pre-synaptic markers (PSD-95 and synaptophysin, respectively) displays the enrichment of PSD-95 and the absence of synaptophysin in the Triton-Insoluble Fraction (TIF). **(D)** Differences in basal intracellular calcium levels are detected in wt vs *Atm^-/-^*. Kruskal-Wallis test followed by Dunn’s multiple comparison test: p<0.0001. Calcium changes induced by exogenous KCl 50mM variations. Number of cells= at least 300 cells/each group. Kruskal-Wallis test followed by Dunn’s multiple comparison test: p=0.0004. Coverslips: wt= 10; *Atm^+/-^*= 14; *Atm^-/-^*=6.

**Supplementary Figure 4. (A)** Representative traces of eIPSCs in wt, *Atm^+/-^* and *Atm^-/-^* cultures and relative analysis of mean eIPSCs amplitude (eIPSCs: Kruskal-Wallis followed by Dunn’s multiple comparison test: p=0.042). Independent experiments = 3**.** **(B)** Representative traces of short-term plasticity experiments performed in wt, *Atm^+/-^* and *Atm^-/-^* neurons and quantification of PPR (A2/A1) at inhibitory synapses (P2/P1: Ordinary One Way Anova followed by Dunnett's multiple comparisons test: p<0.0001). Independent experiments = 3.

**Supplementary Figure 5. (A)** Left panel: representative Western Blotting lanes. Right panel: analysis on hippocampal tissues from wt mice treated in the prenatal life with VPA (or sal) and in adulthood with intranasal KU (or DMSO) show comparable levels of Gluk-5 and Gluk-1 KARs subunits (Gluk-5: Ordinary One Way Anova followed by Tukey's multiple comparisons test: p=0.23, Gluk-1: Ordinary One Way Anova followed by Tukey's multiple comparisons test: p=0.26). **(B)** Western Blotting experiments performed in wt and *Atm^+/-^* hippocampi show significant differences in Gluk-5 levels. Gluk-5 expression remains unchanged in hippocampi of *Atm^+/-^*mice treated with Fluox (Gluk-5: Ordinary One Way Anova followed by Kruskal-Wallis test: p=0.0002).

Supplementary table 1. Differentially expressed gene in the hippocampus of KU55933 vs. vehicle- treated mice. Table showing base means across samples, log2 fold changes, P-values and adjusted P-Values. Adjusted P-Values cut-off P <0.05.

Supplementary table 2. Detailed GO-enrichment analysis of differentially expressed genes in the hippocampus of KU55933 vs. vehicle- treated mice.

**Supplementary Material and methods**

**Novel Object Recognition (NOR) test**

The test was conducted over a four-day period in an open plastic arena (60 x 50 x 30 cm), as previously described (23). Animals were habituated to the test arena for 10 min on the first day. After 3-day habituation, mice were subjected to familiarization (T1) and novel object recognition (T2). During the initial familiarization stage, two identical objects were placed in the centre of the arena equidistant from the walls and from each other. Each mouse was placed in the centre of the arena between the two objects for a maximum of 10 min or until it had completed 30 s of cumulative object exploration. Object recognition was scored when the animal was within 0.5 cm of an object with its nose toward the object. Exploration was not scored if a mouse reared above the object with its nose in the air or climbed on an object. Mice were returned to the home cage after familiarization and retested 180 min later, and in the arena a novel object (never seen before) took the place of one of the two familiar. Scoring of object recognition was performed in the same manner as during the familiarization phase. From mouse to mouse the role (familiar or new object) as well as the relative position of the two objects were counterbalanced and randomly permuted. The objects for mice to discriminate consisted of coloured plastic cylinders and coloured plastic Lego stacks of different shape. The arena was cleaned with 70% ethanol after each trial. The basic measure was the time (in seconds) taken by the mice to explore the objects in the two trials. The performance was evaluated by calculating a discrimination index (N-F/N+F), where n= time spent exploring the new object during T2, F= time spent exploring the familiar object during T2 (54).

**Immunohistochemical Staining**

P40 male and female animals were euthanized, and the brains were removed and fixed in 4% paraformaldehyde for 48 hours. Brains were then included in 4% low melting point agarose (MilliporeSigma) in 1× PBS. After agarose polymerization sections of 50 μm thickness were obtained using a VT1000S vibratome (Leica Microsystems). Immunofluorescence staining was carried out on free-floating sections at the level of dorsal hippocampus. Staining was performed using a primary antibody previously described for in vitro staining. Images were examined by means of a confocal laser scanning microscope. Single plane images were acquired in the stratum radiatum of the CA1 subfield of the hippocampus using 40x oil immersion lens with an additional electronic zoom factor of up to 2. VGLUT and VGAT area were calculated as the total area of the positive puncta within a selected Region Of Interest (ROI), normalized to the total area of the ROI. At least two regions were analysed for each section and at least five sections were used for each animal.
