# Supplementary material for: ATM rules neurodevelopment and glutamatergic transmission in the hippocampus but not in the cortex
Source: Cell Death Dis. 2022 Jul 16;13(7):616. doi: 10.1038/s41419-022-05038-7 (PMC9288428; doi:10.1038/s41419-022-05038-7)

Fig 1. Fluox prenatal delivery: HIPPO

Actin

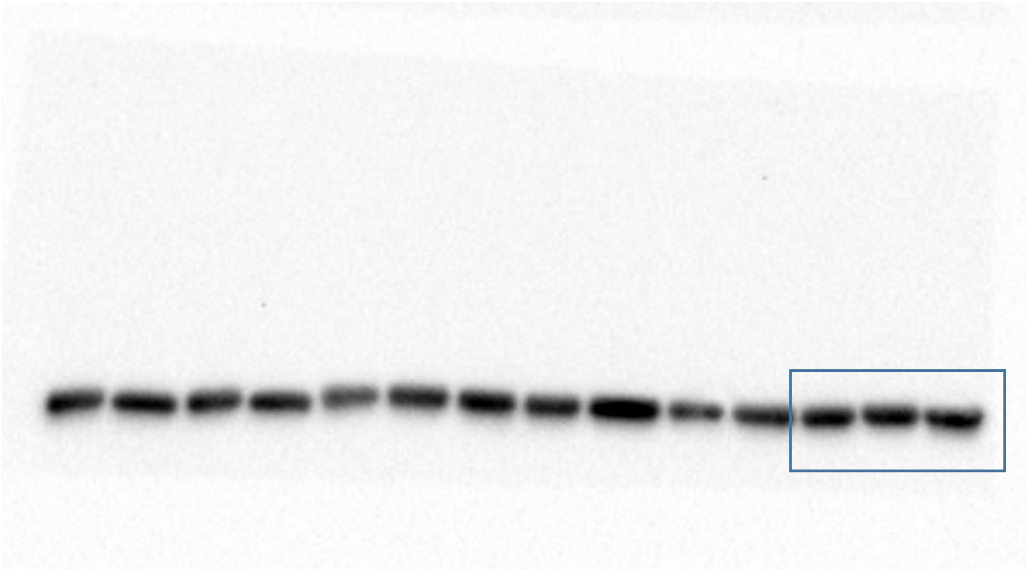

KCC2

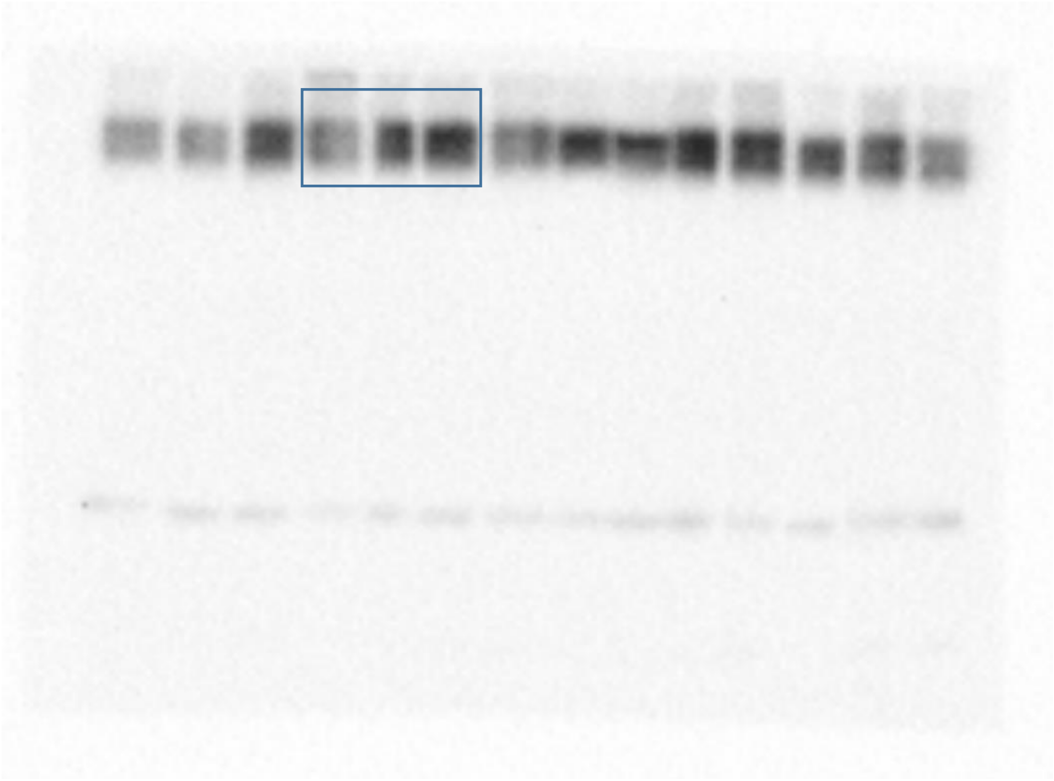

MeCp2

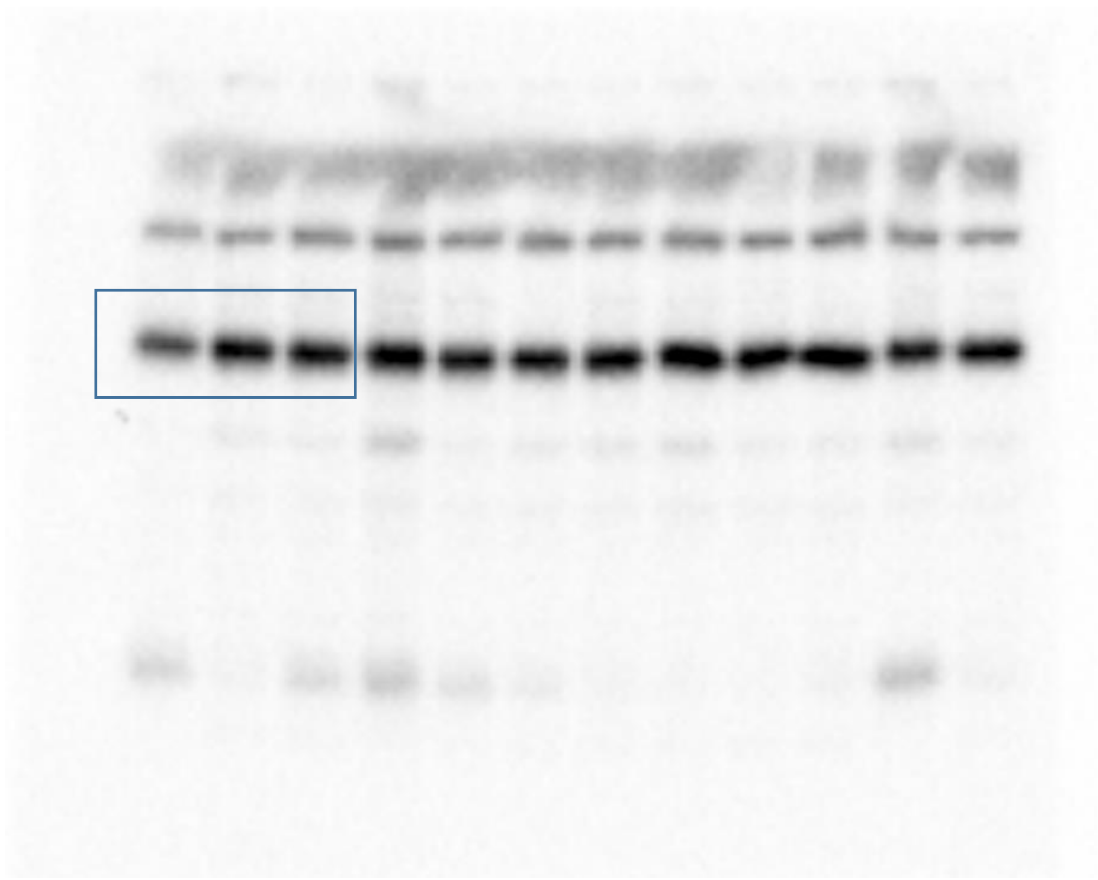

NKCC1

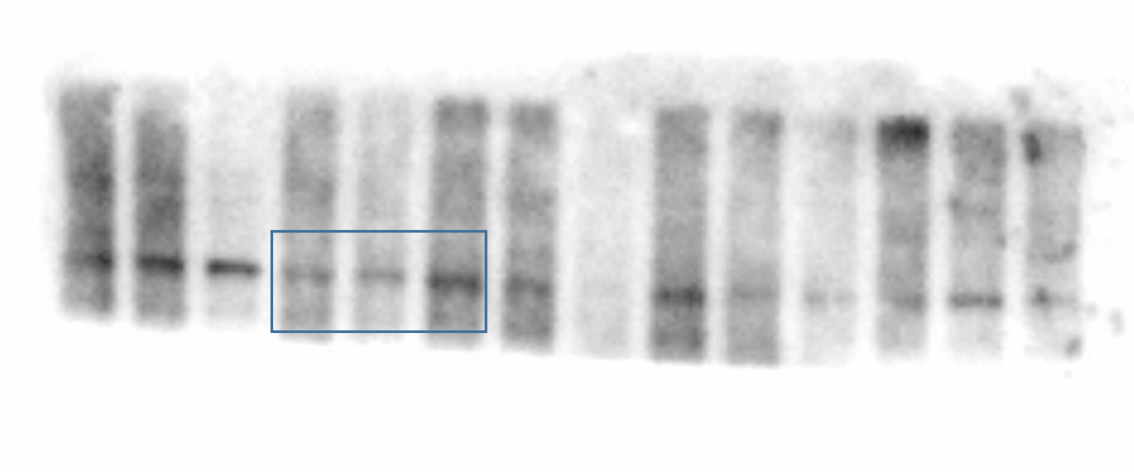

PSD95

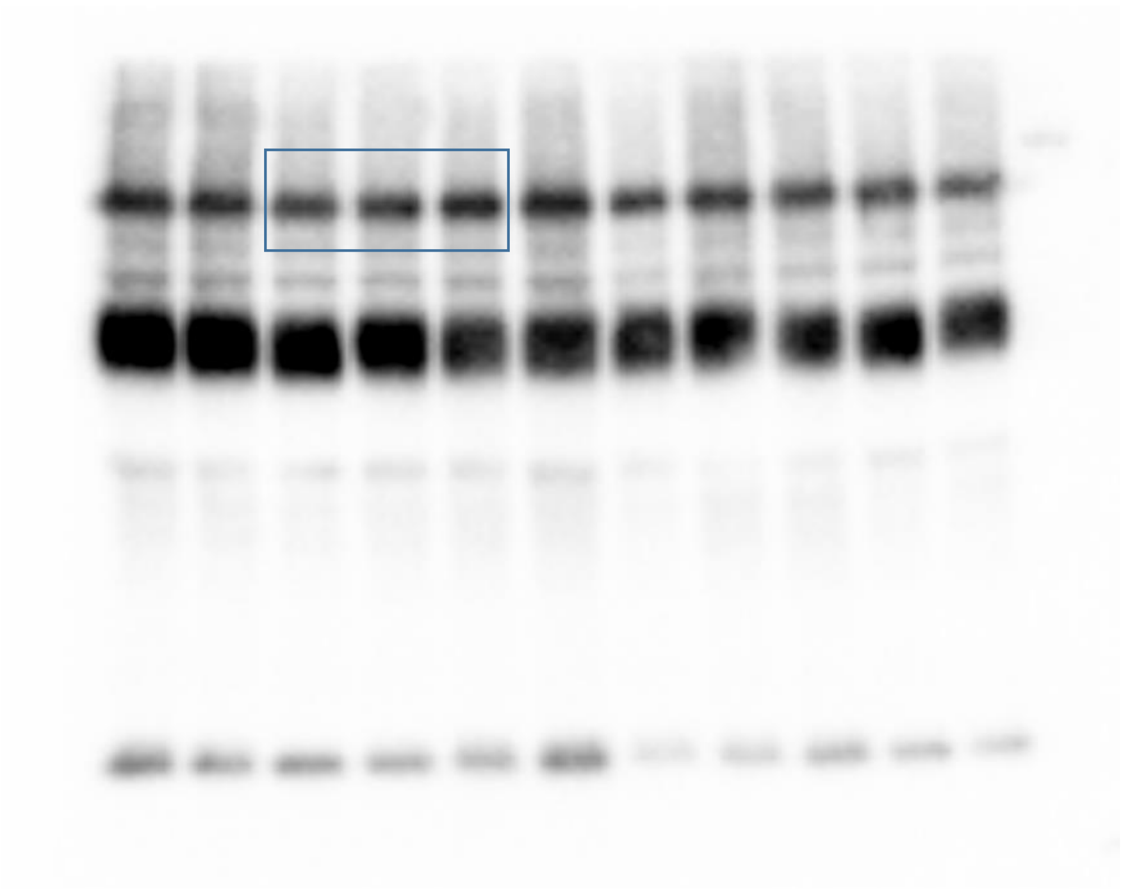

SNAP-25

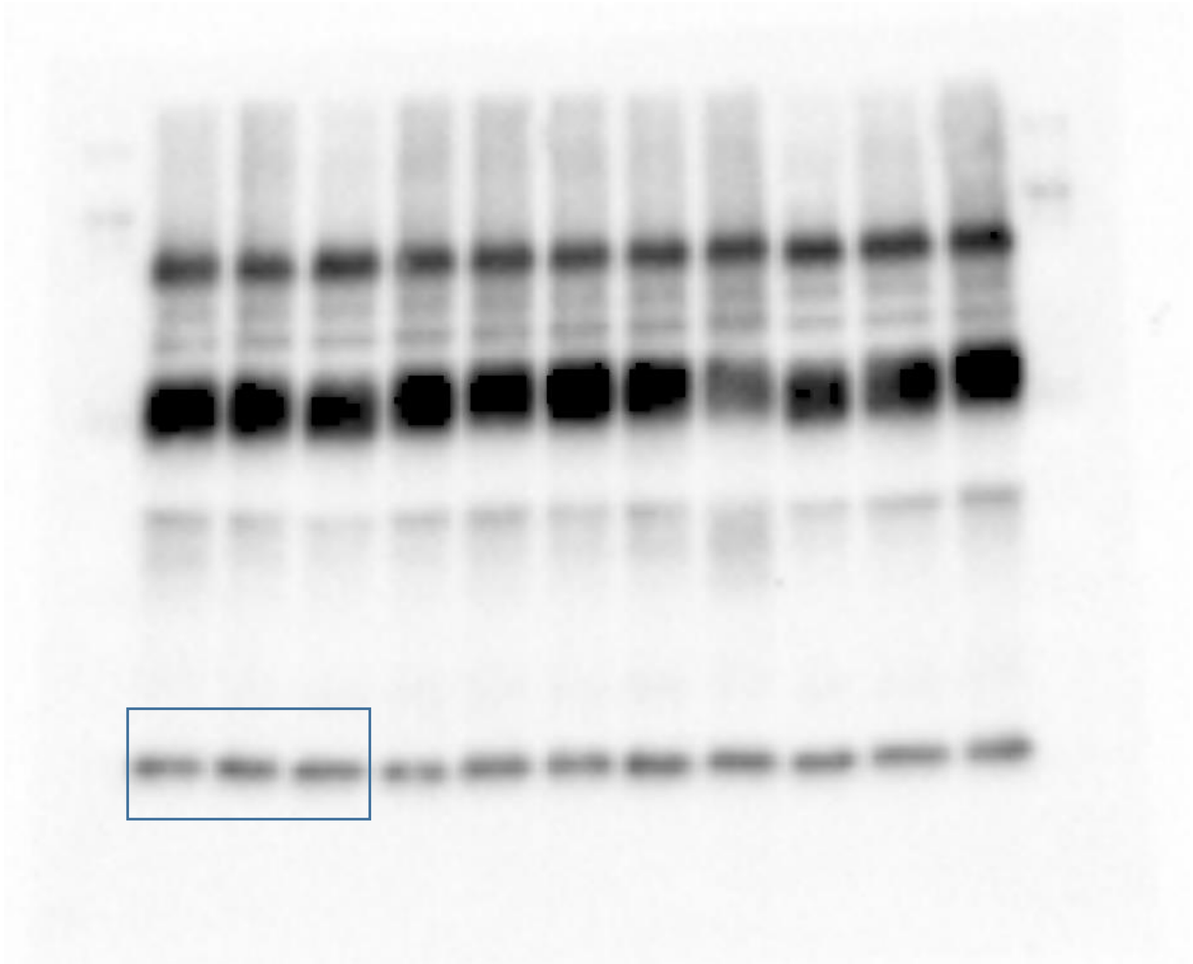

vGlut1

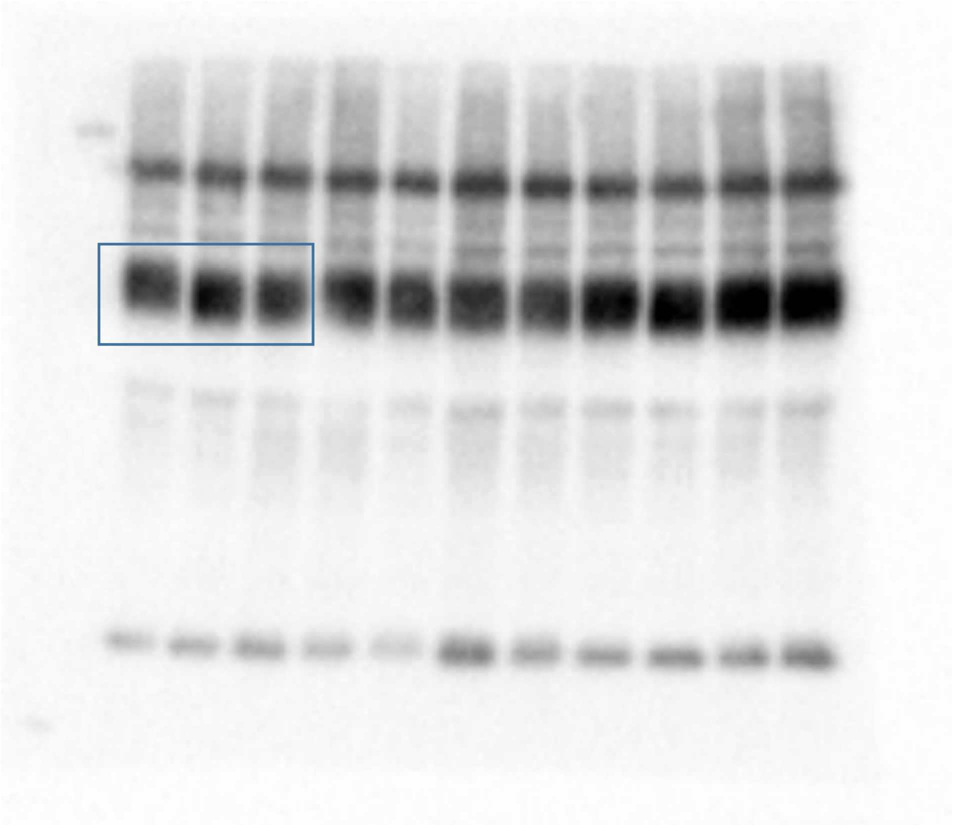

vGAT

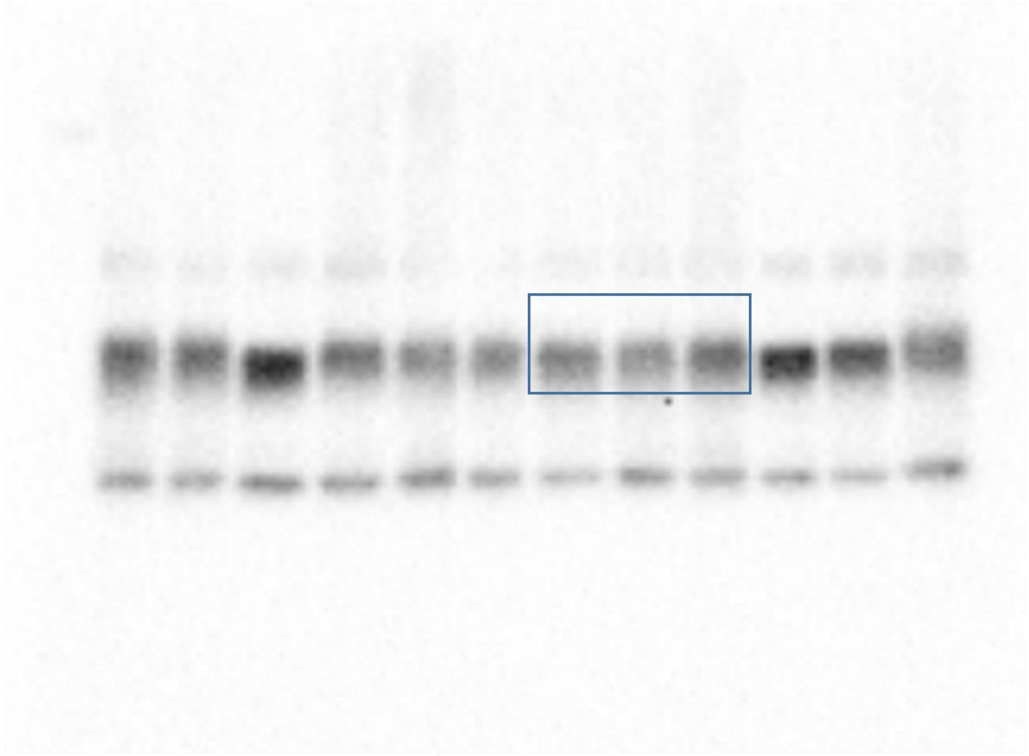

Fig 1. Fluox prenatal delivery: CTX

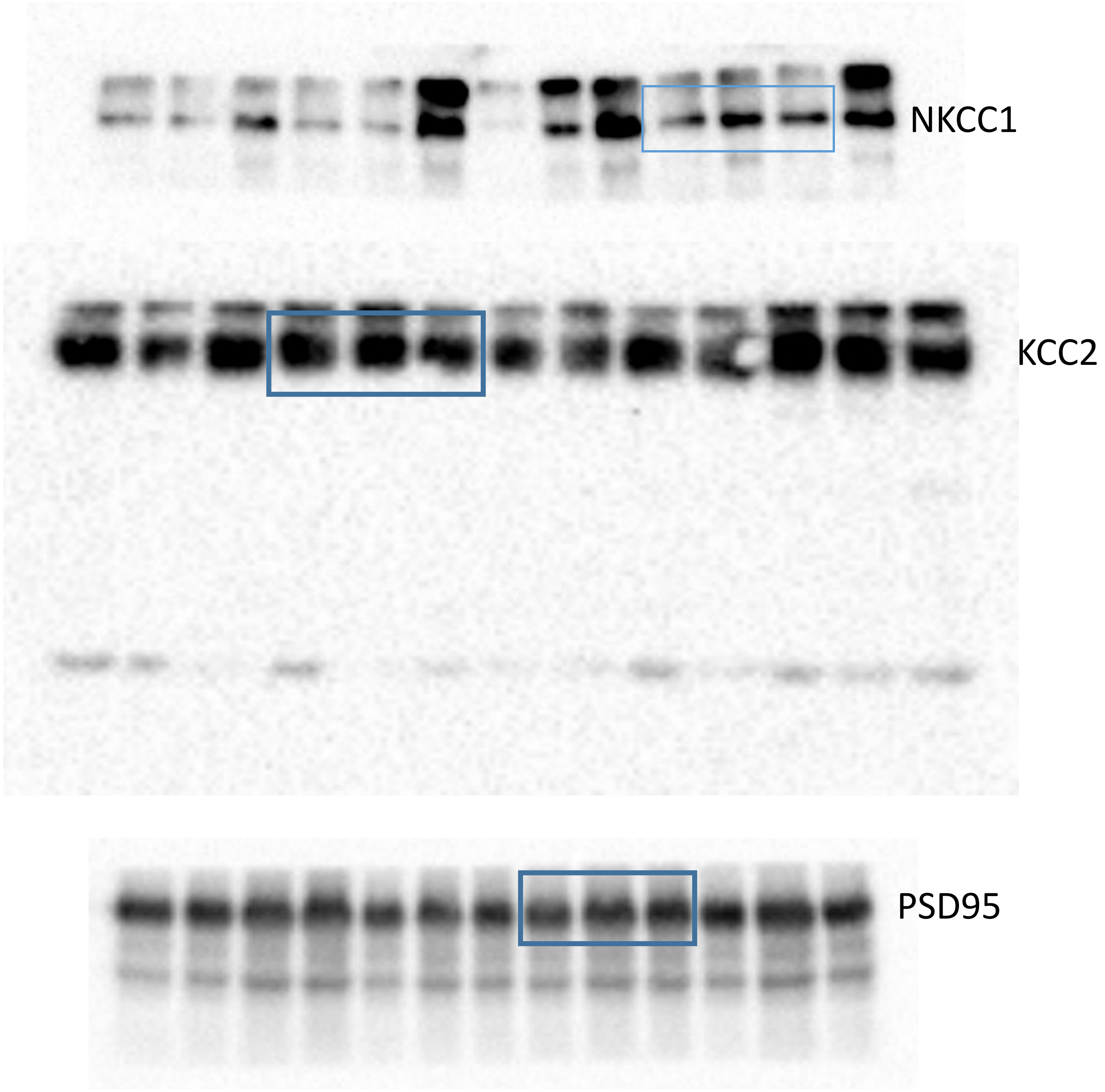

Fig 1. Fluox prenatal delivery: CTX

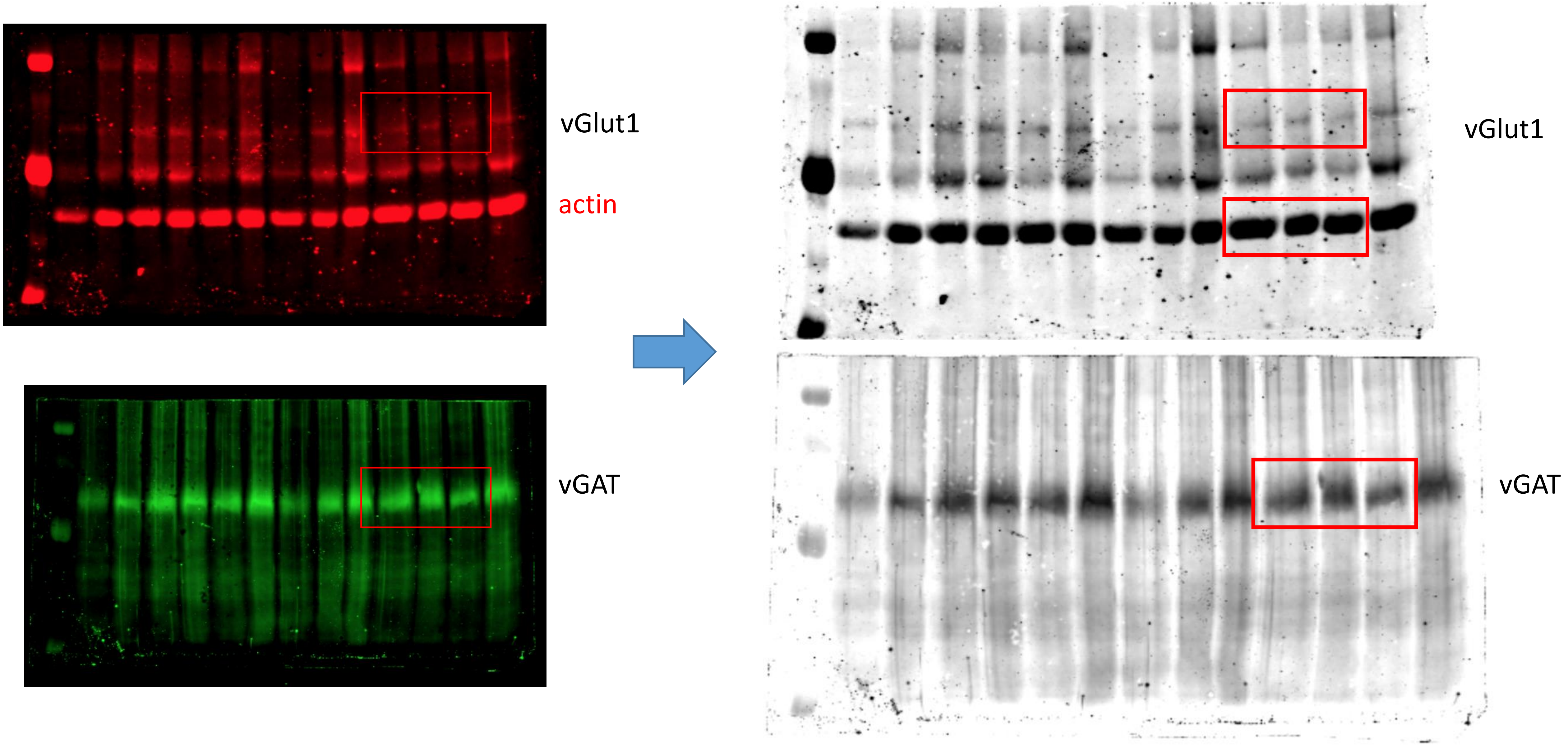

Fig 1. Fluox prenatal delivery: CTX

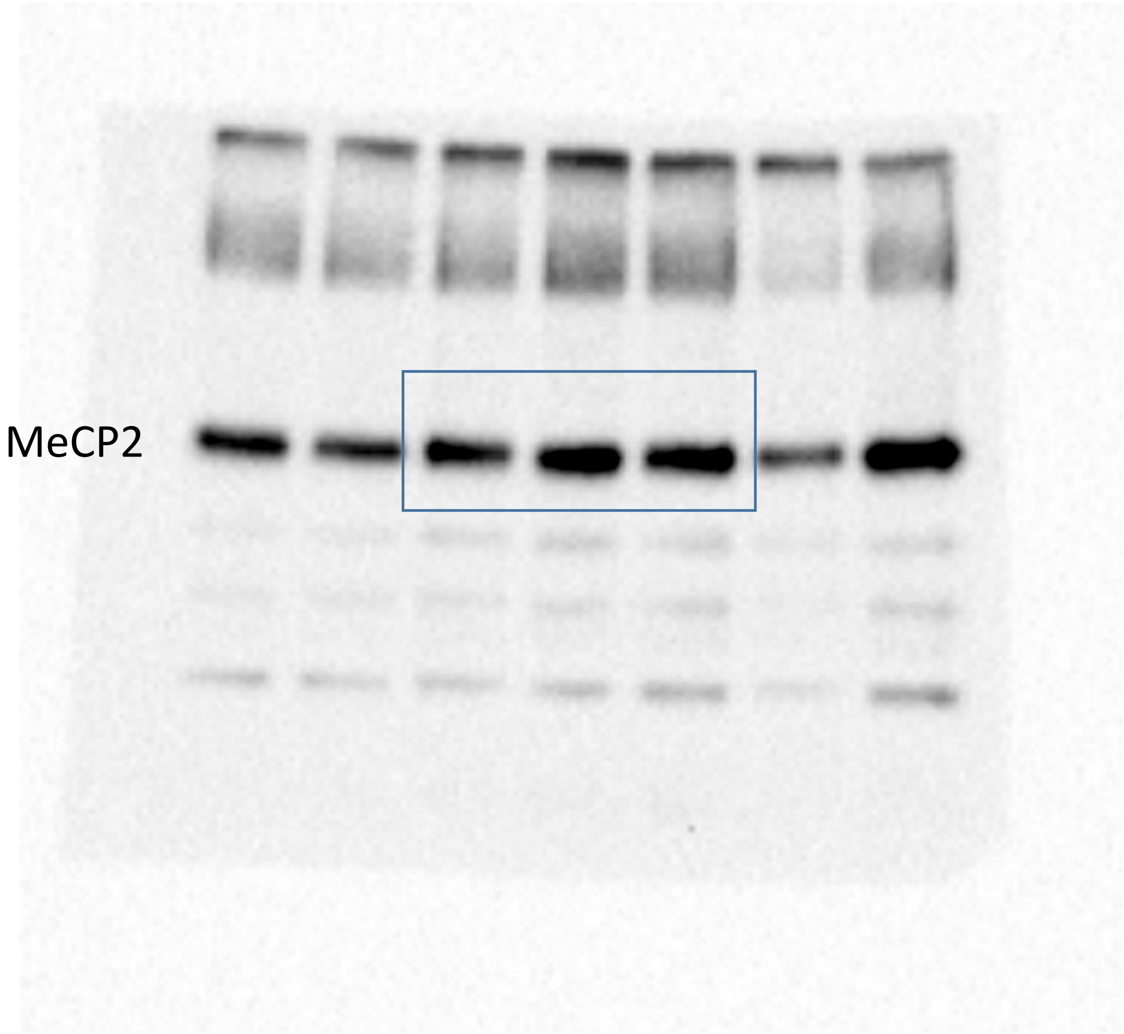

Fig 1. Fluox prenatal delivery: CTX

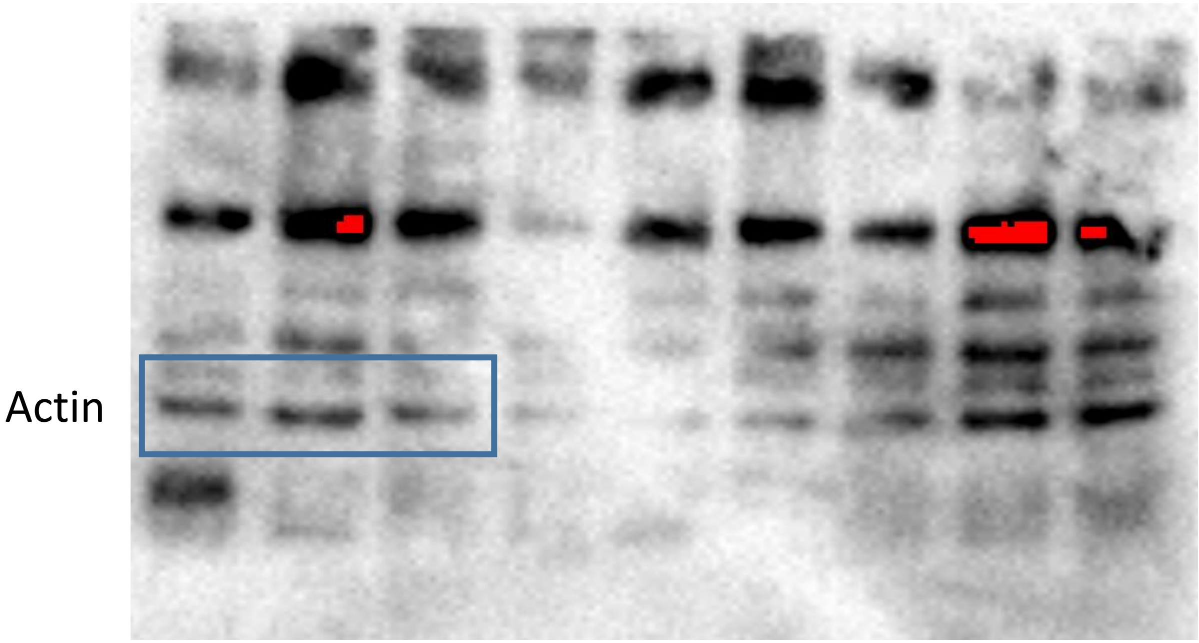

Fig 2C : KCC2 and Actin

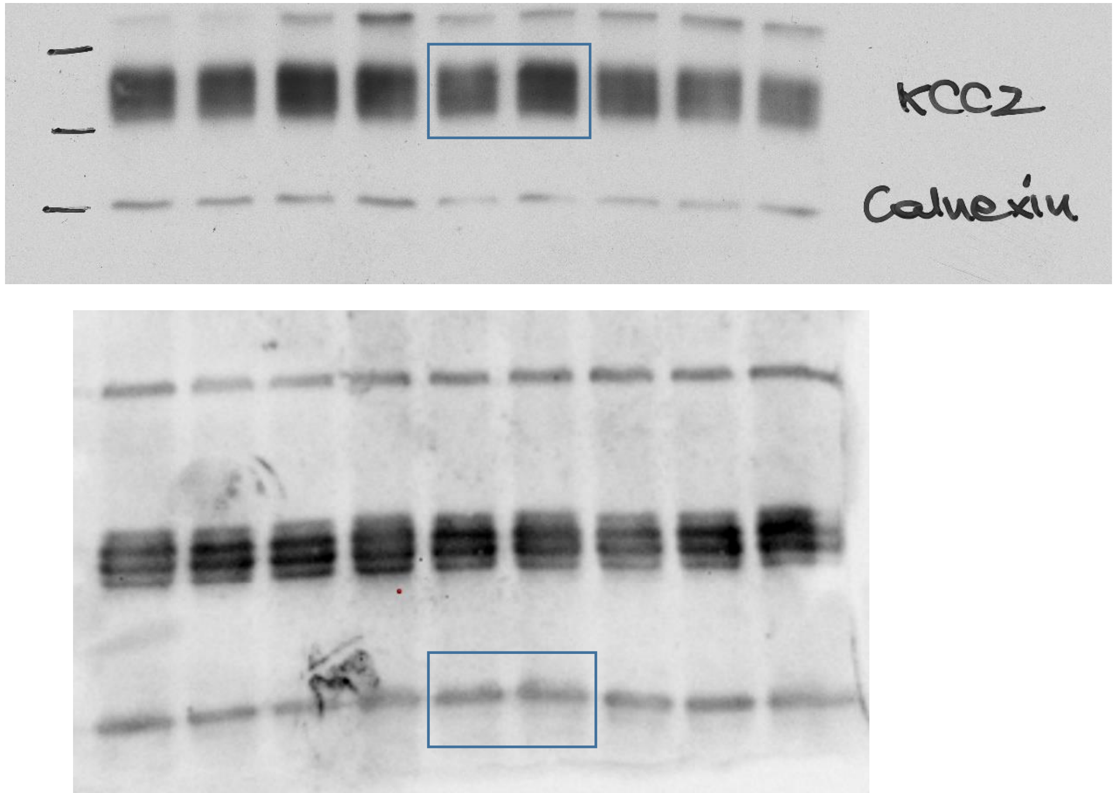

Fig 2 D: Mecp2 and Actin

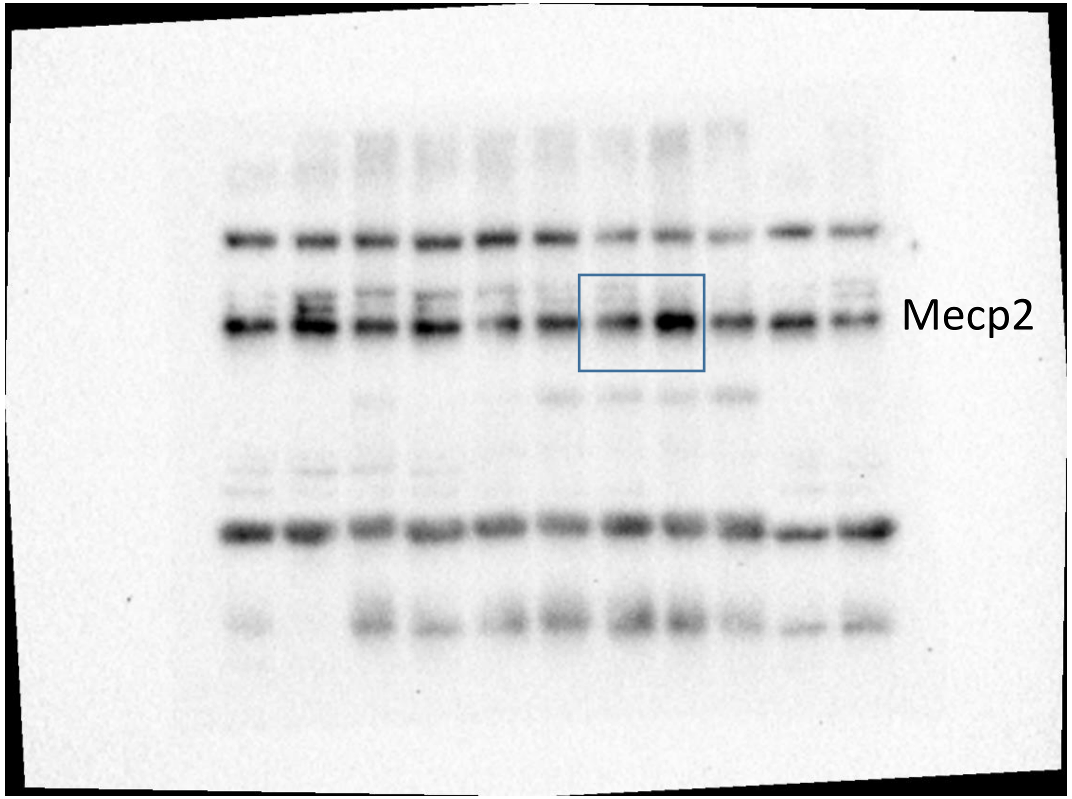

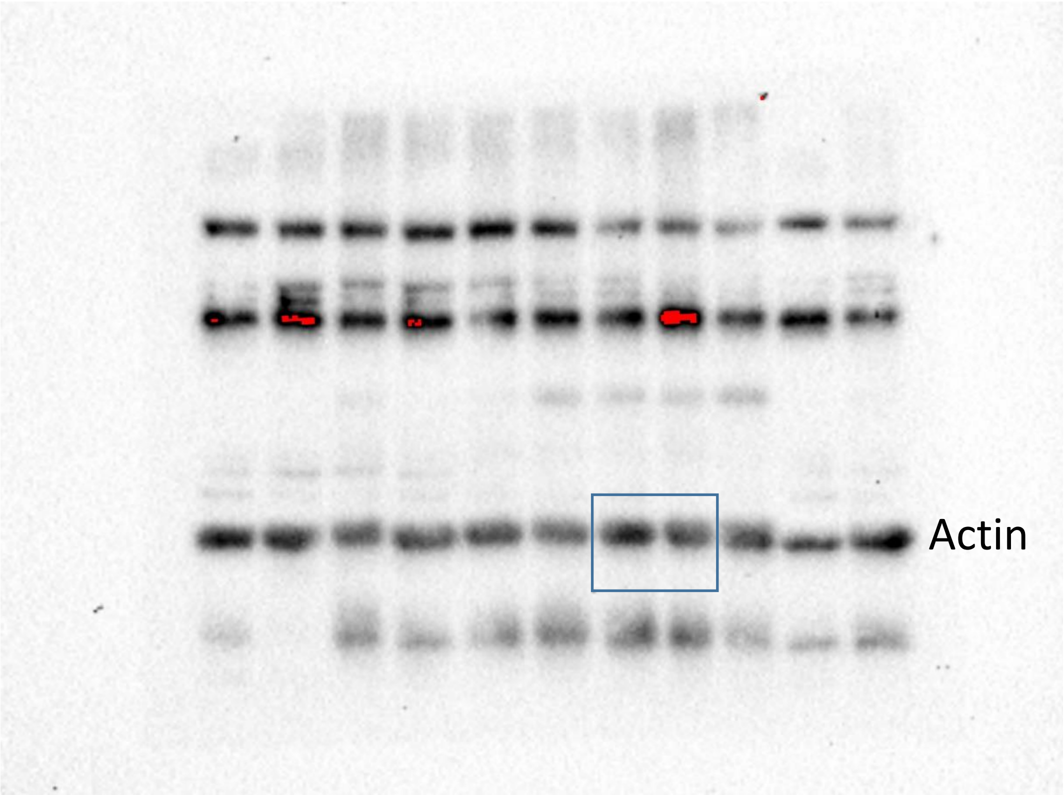

Fig 2 G: KCC2 and Actin

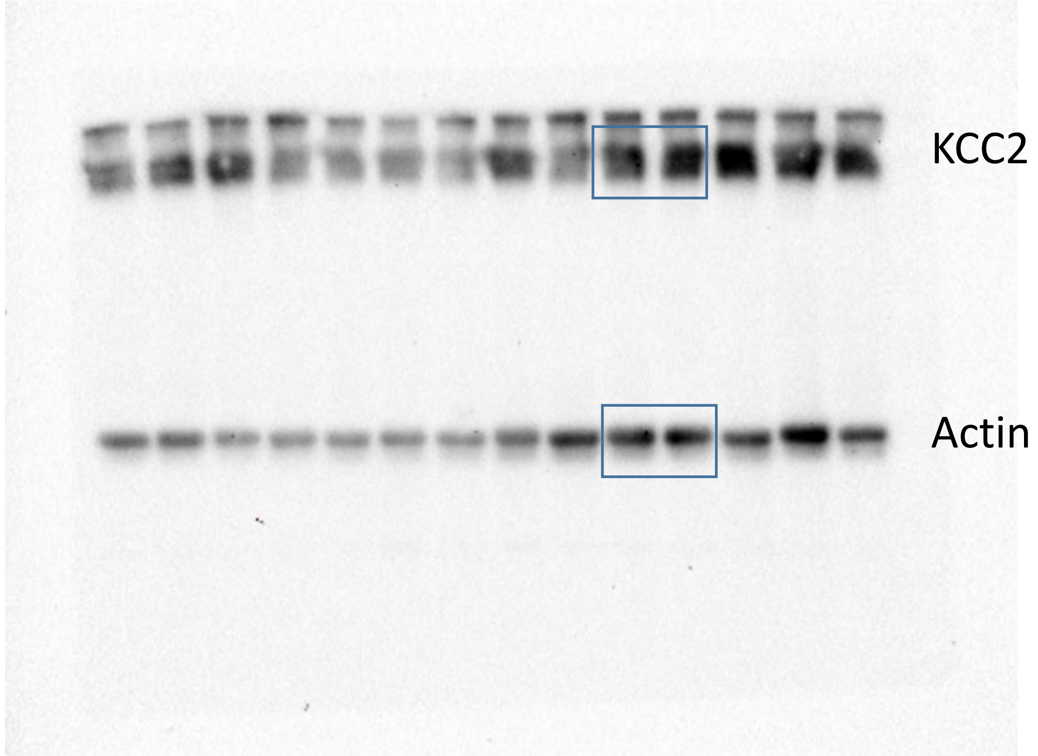

Fig 2 H: Mecp2 and Actin

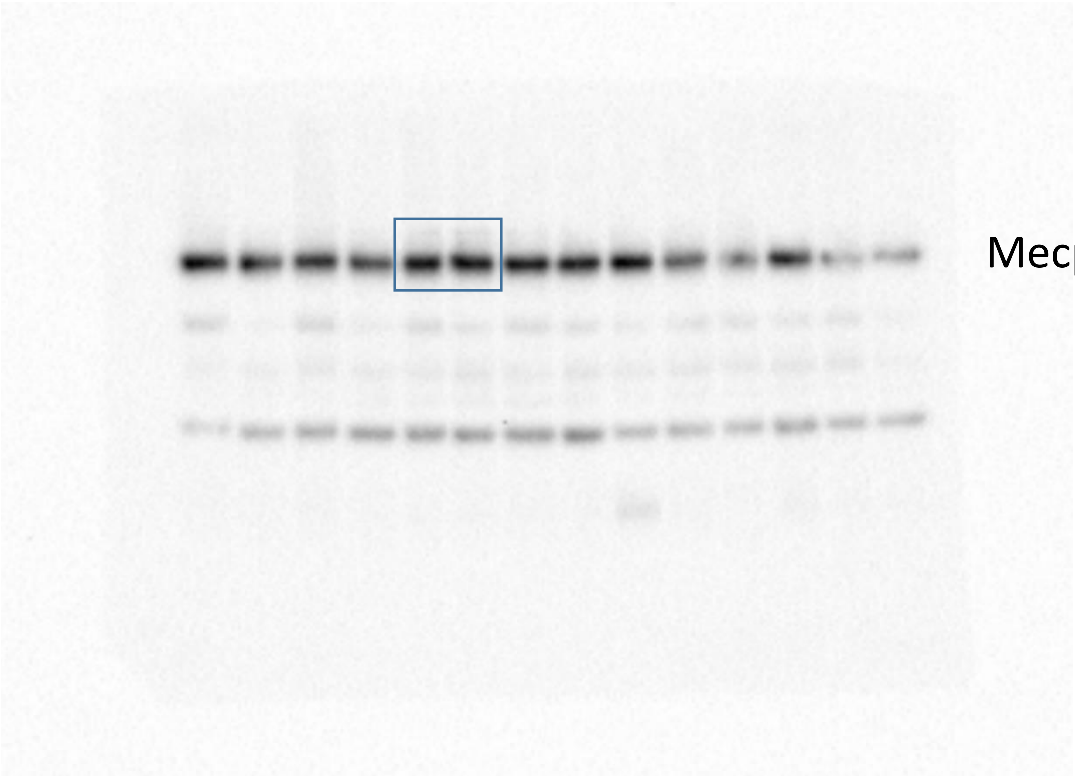

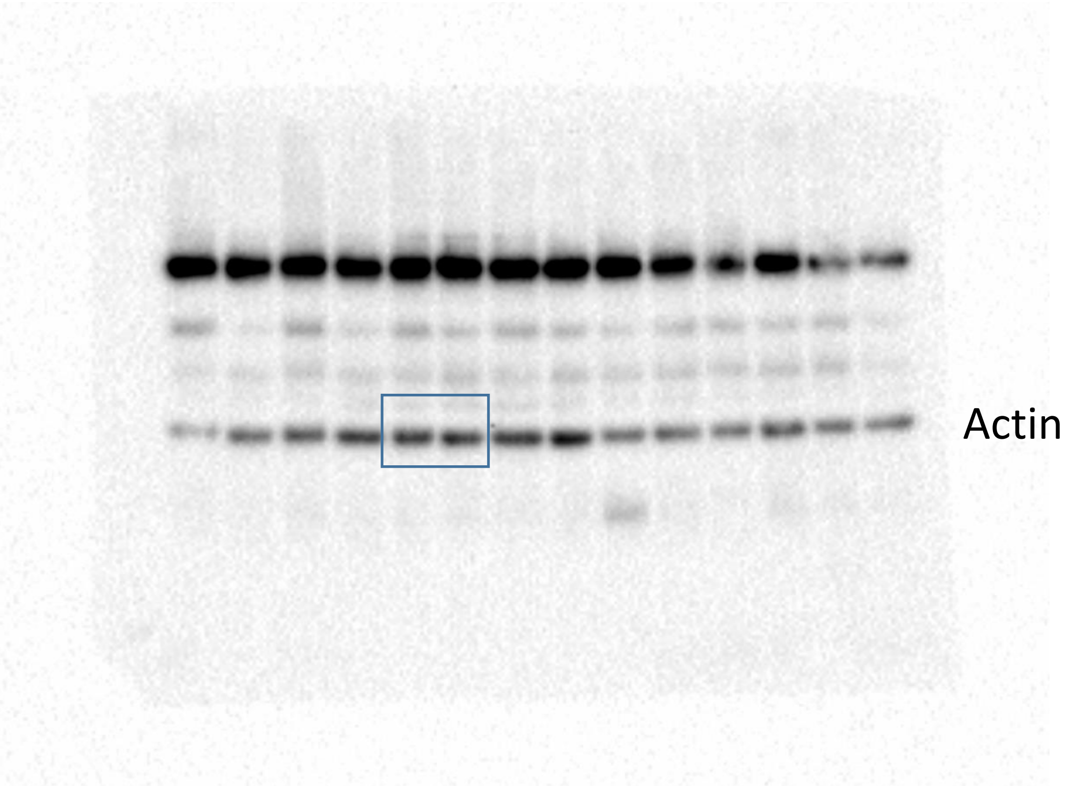

Fig 2 K: KCC2 and Actin

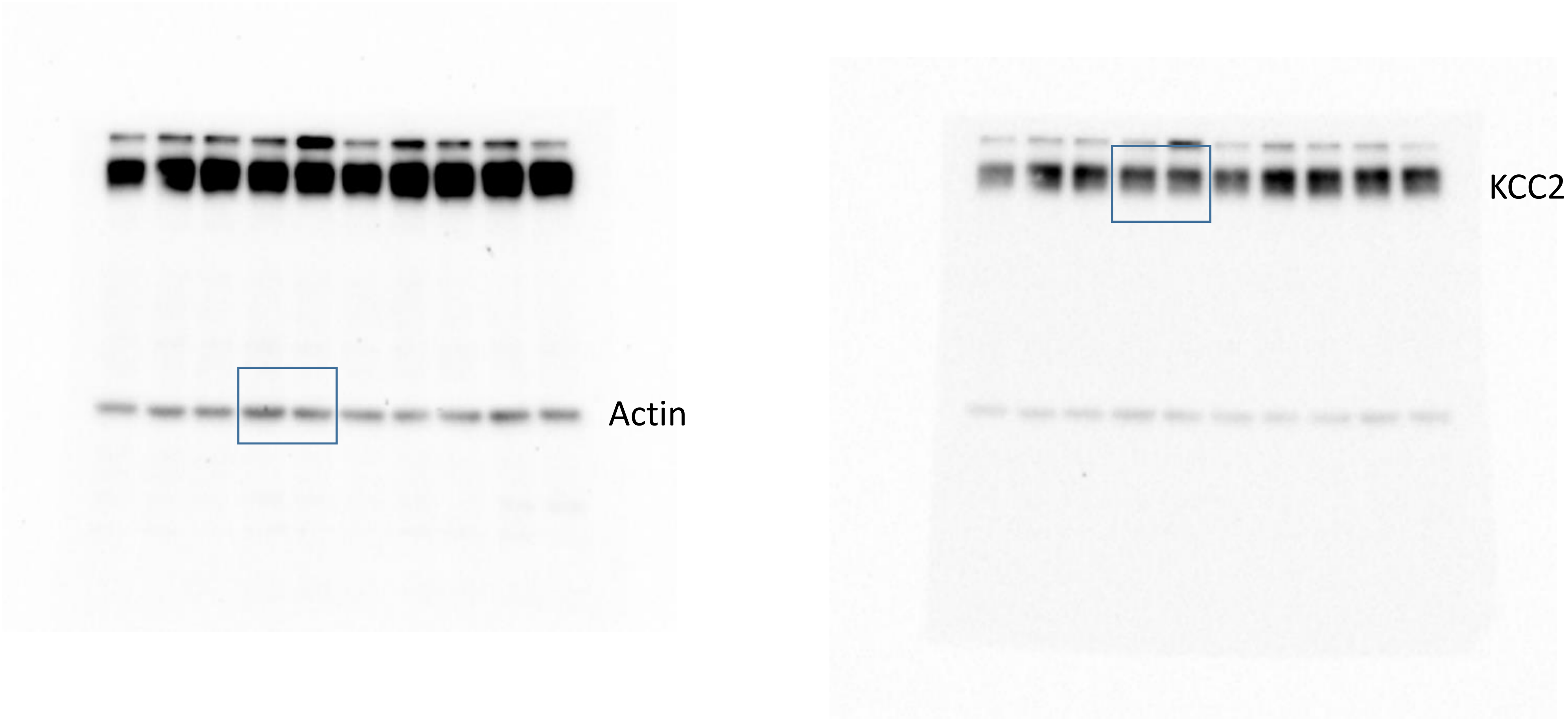

Fig 2 L: Mecp2 and Actin

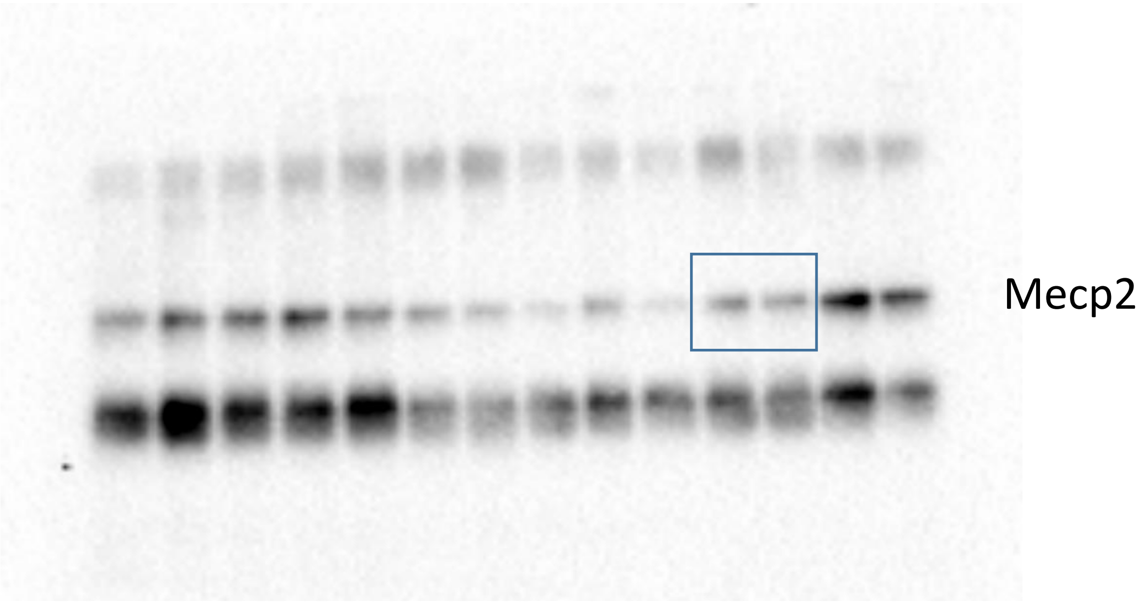

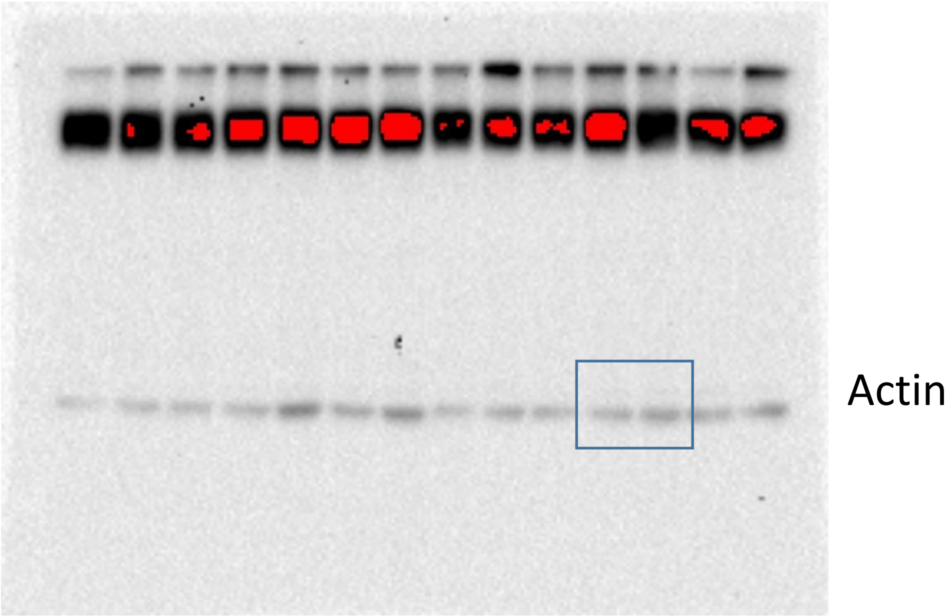

Fig 4 C: KA2-Gluk5 e Actin: hippo

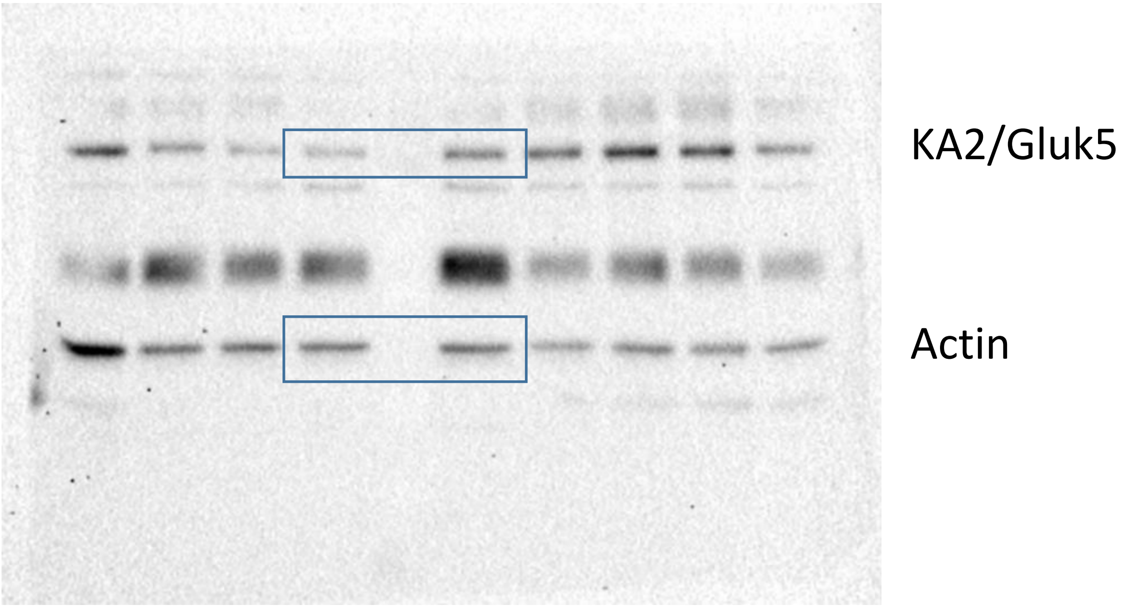

Fig 4 C: Gluk1 e Actin: hippo

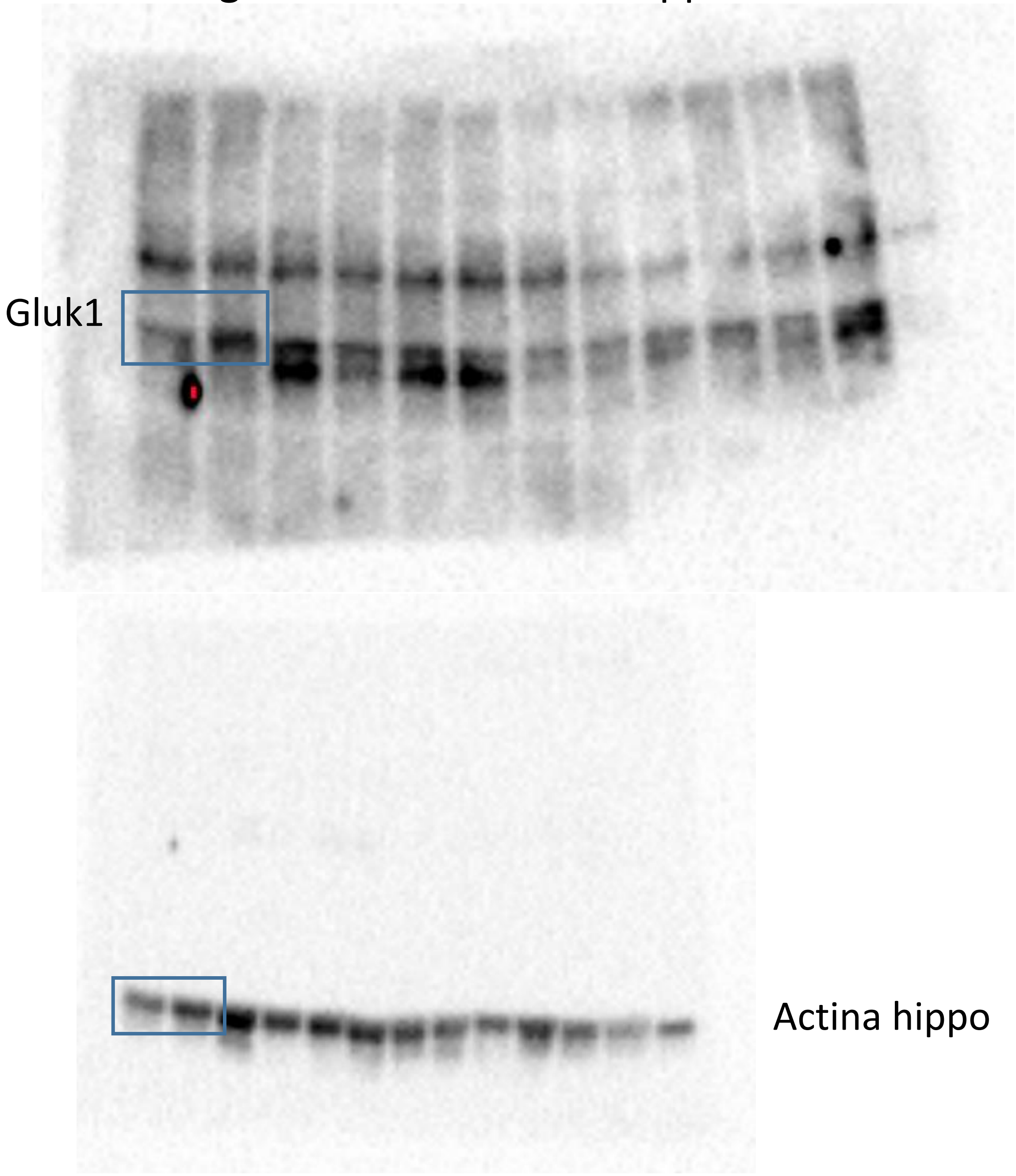

Fig 4 C, KA2-Gluk5 and actin : ctx

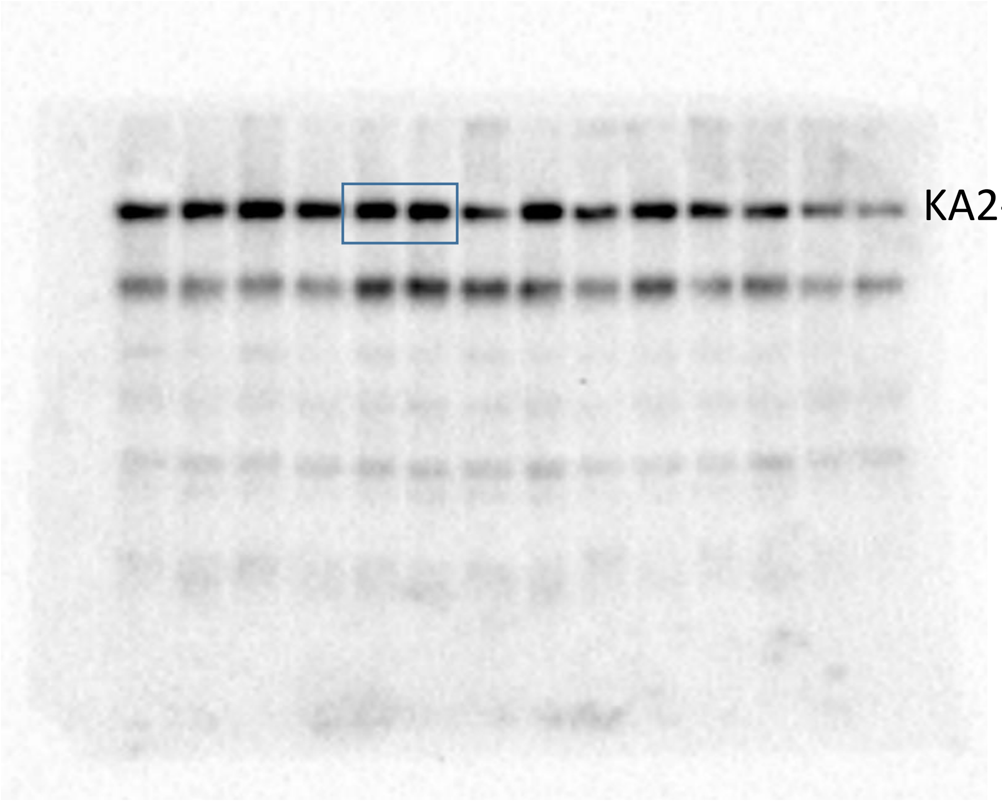

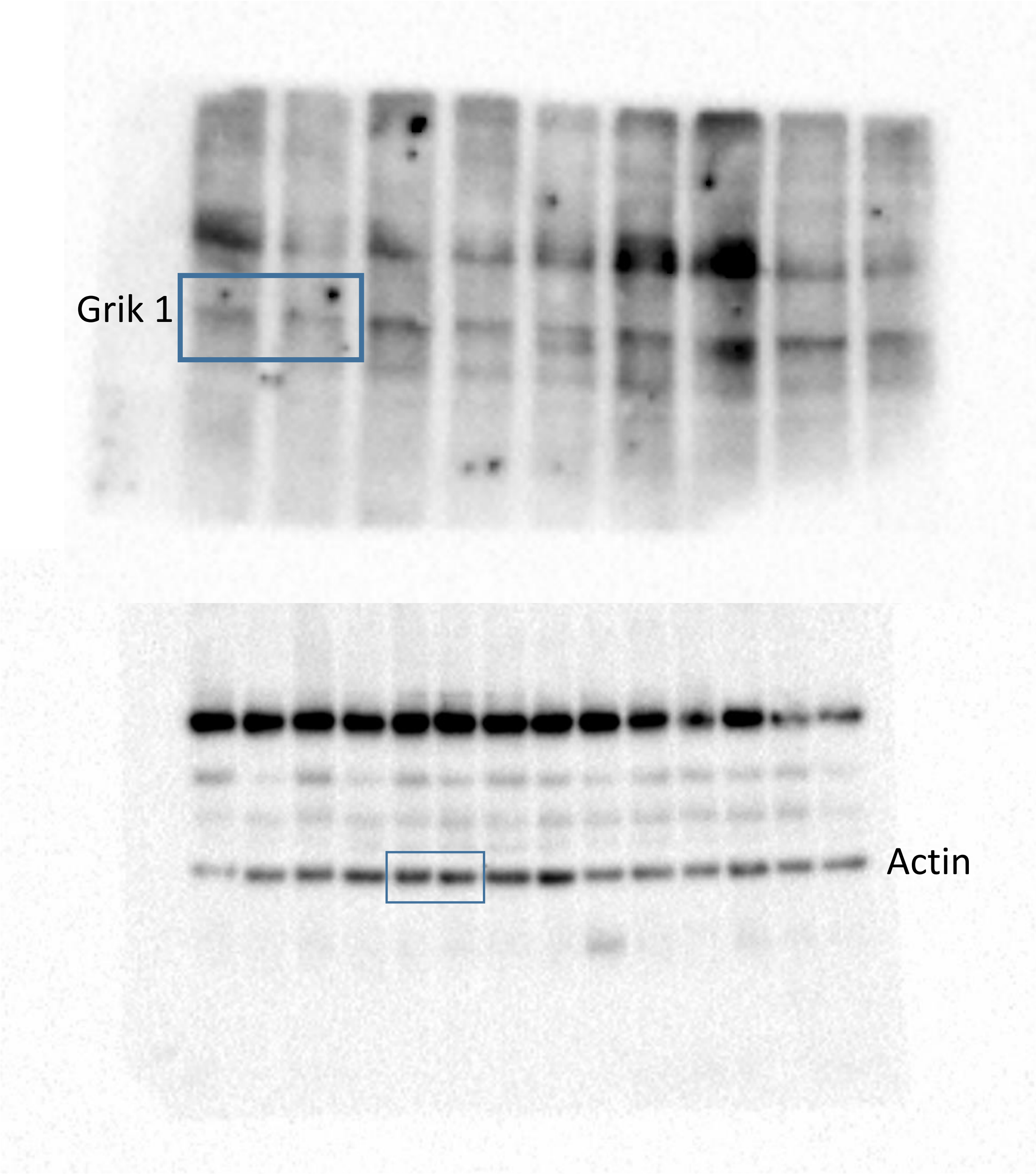

Fig 5D: KA2 and Actin

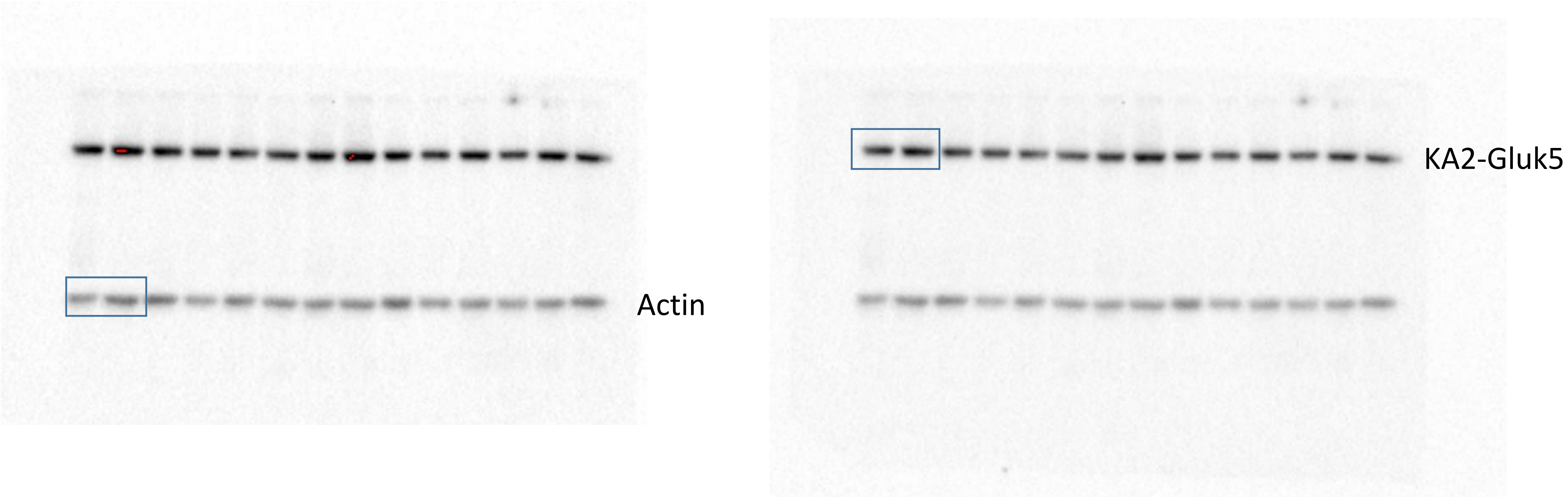

Fig 5E: KA and Actin

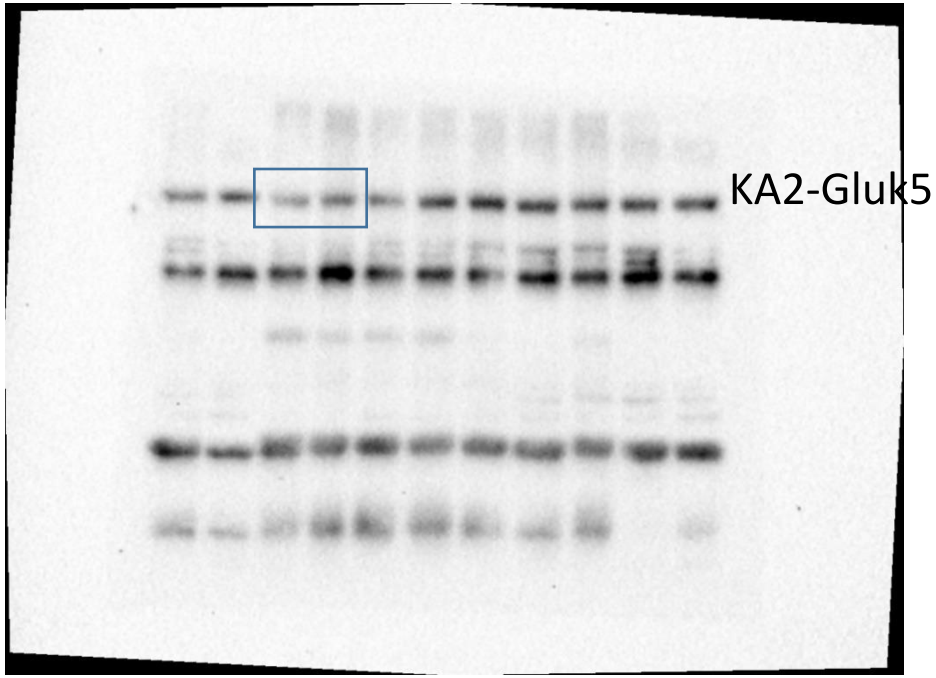

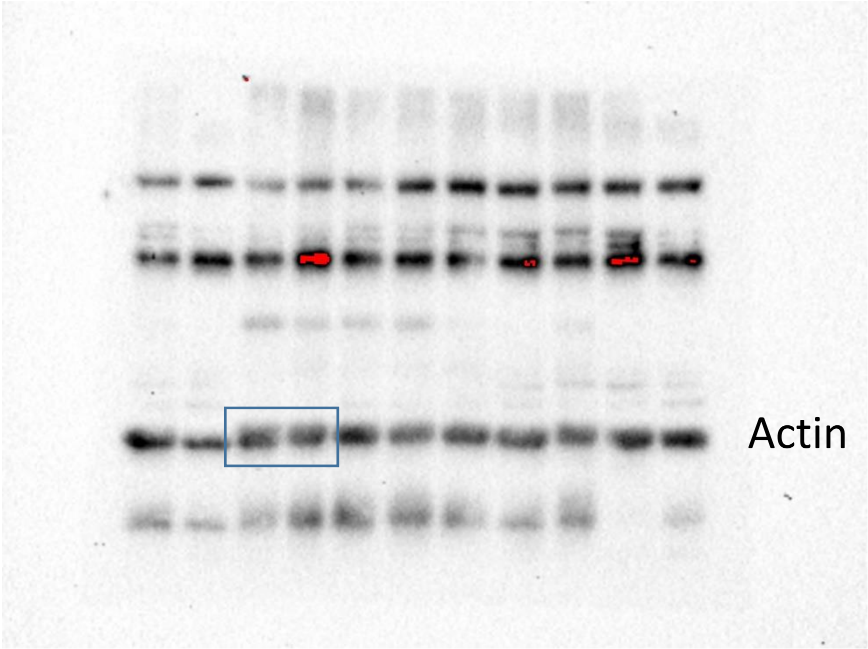

Fig 5C

GluN2A

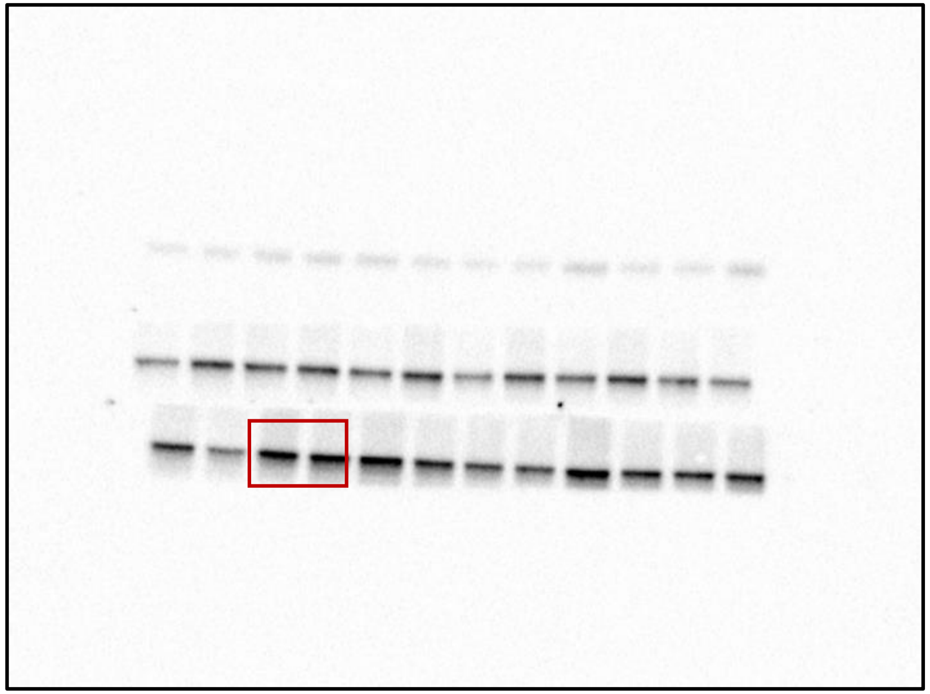

GluN2B

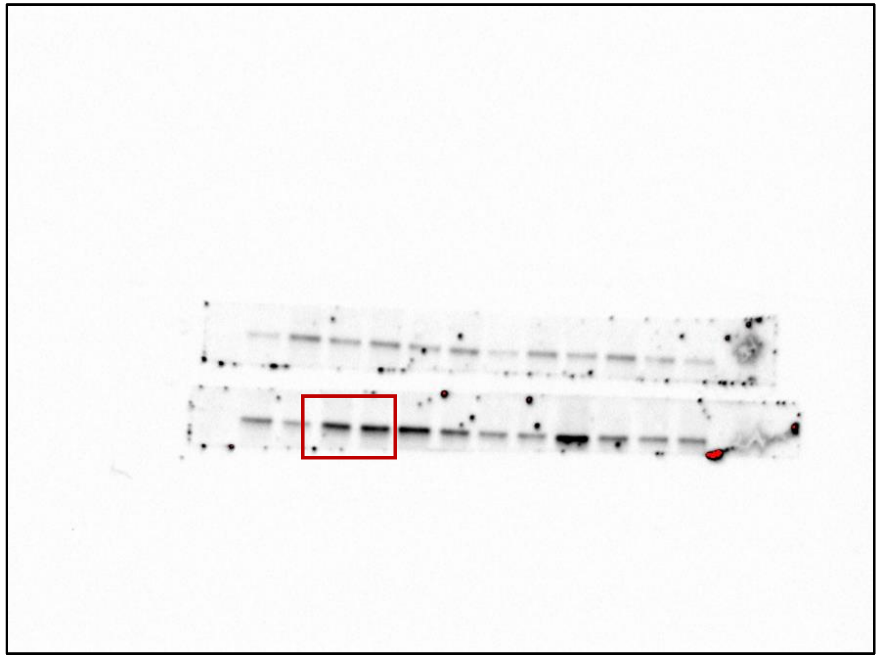

Fig 5C

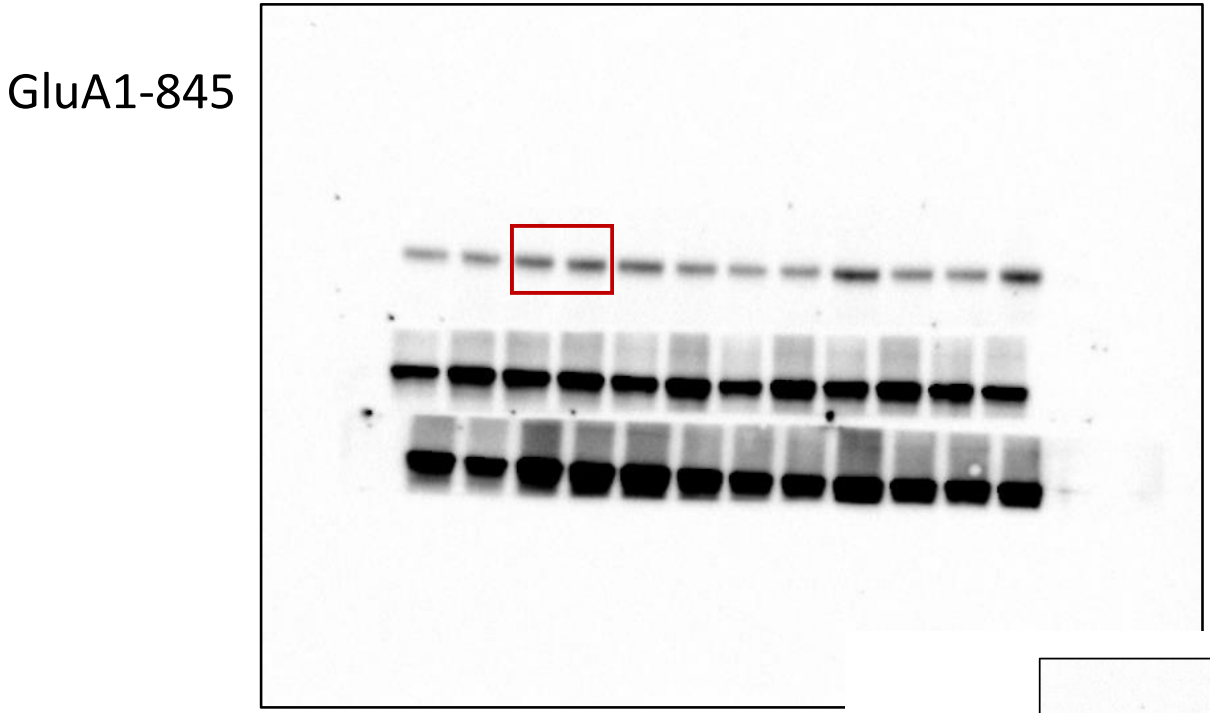

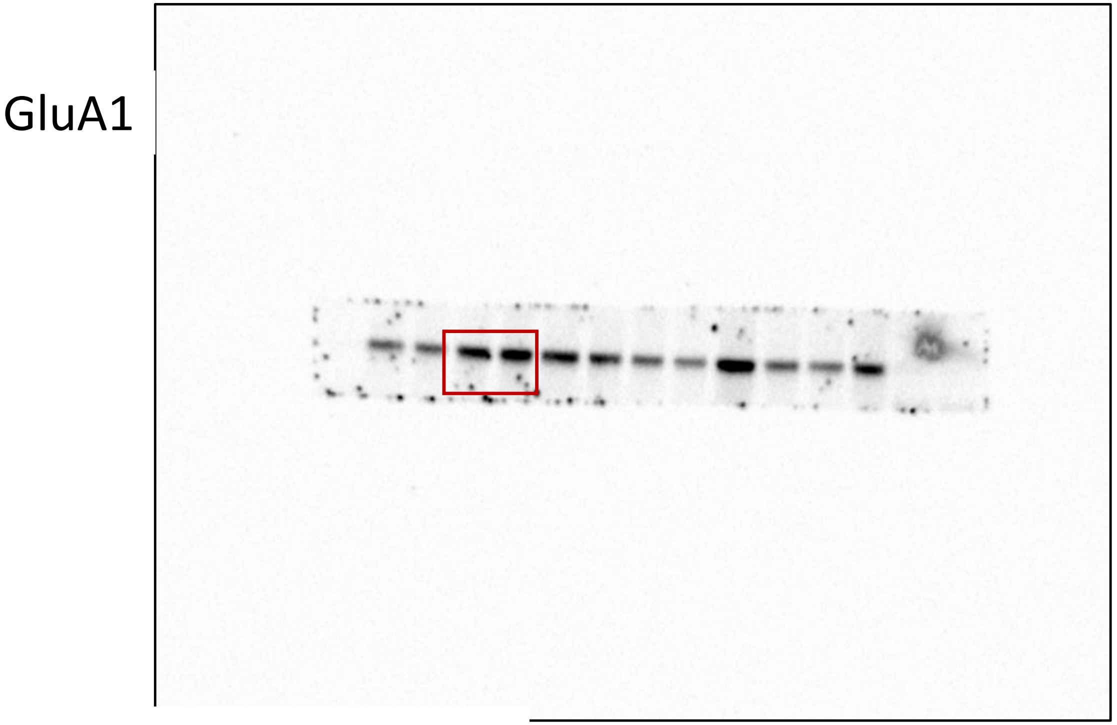

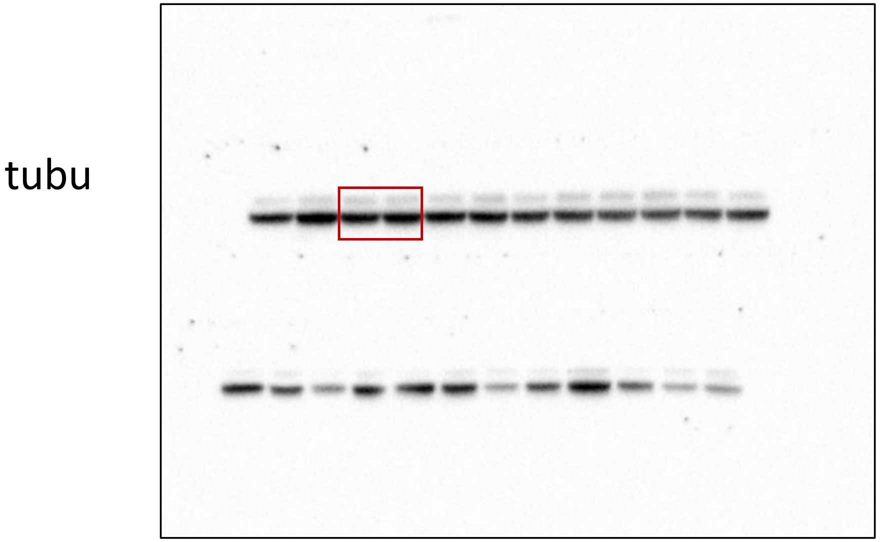

Fig 5C

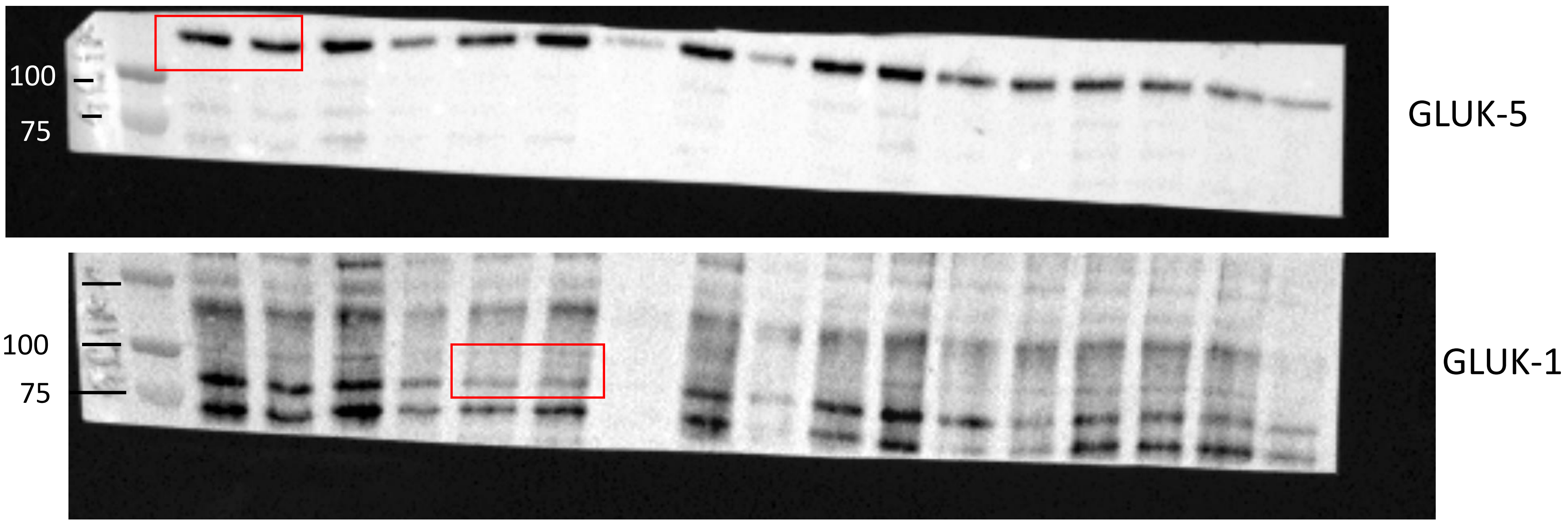

Suppl fig 2A

NKCC1

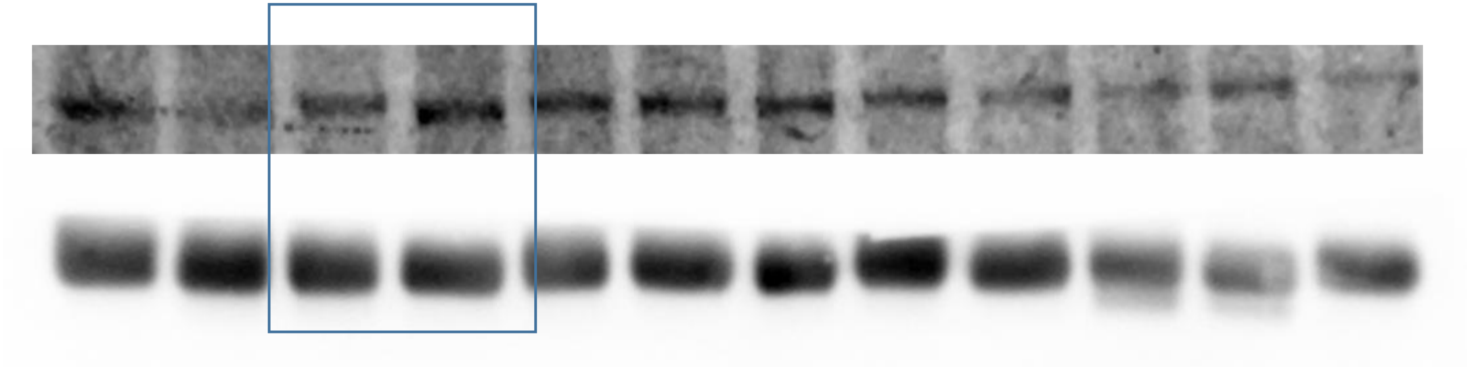

KCC2

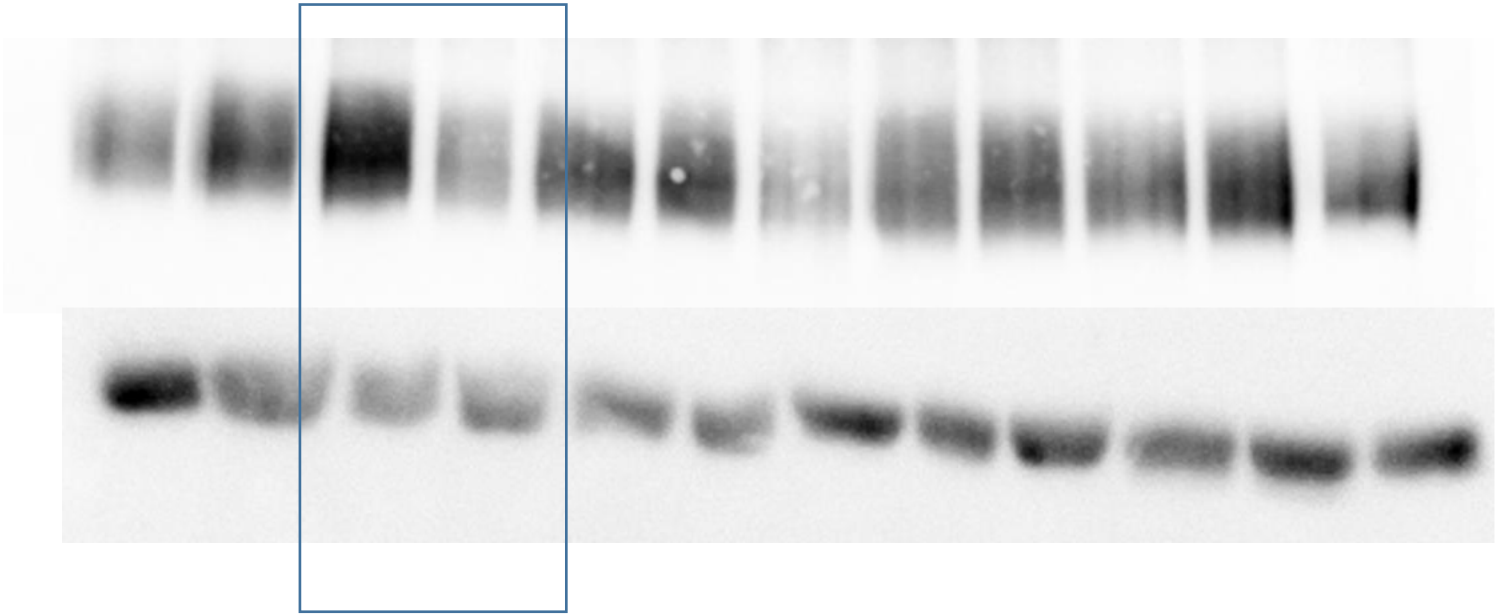

BLOT : TIF CONTROL EXPERIMENT (REVISION, SUPPL FIG 4A)

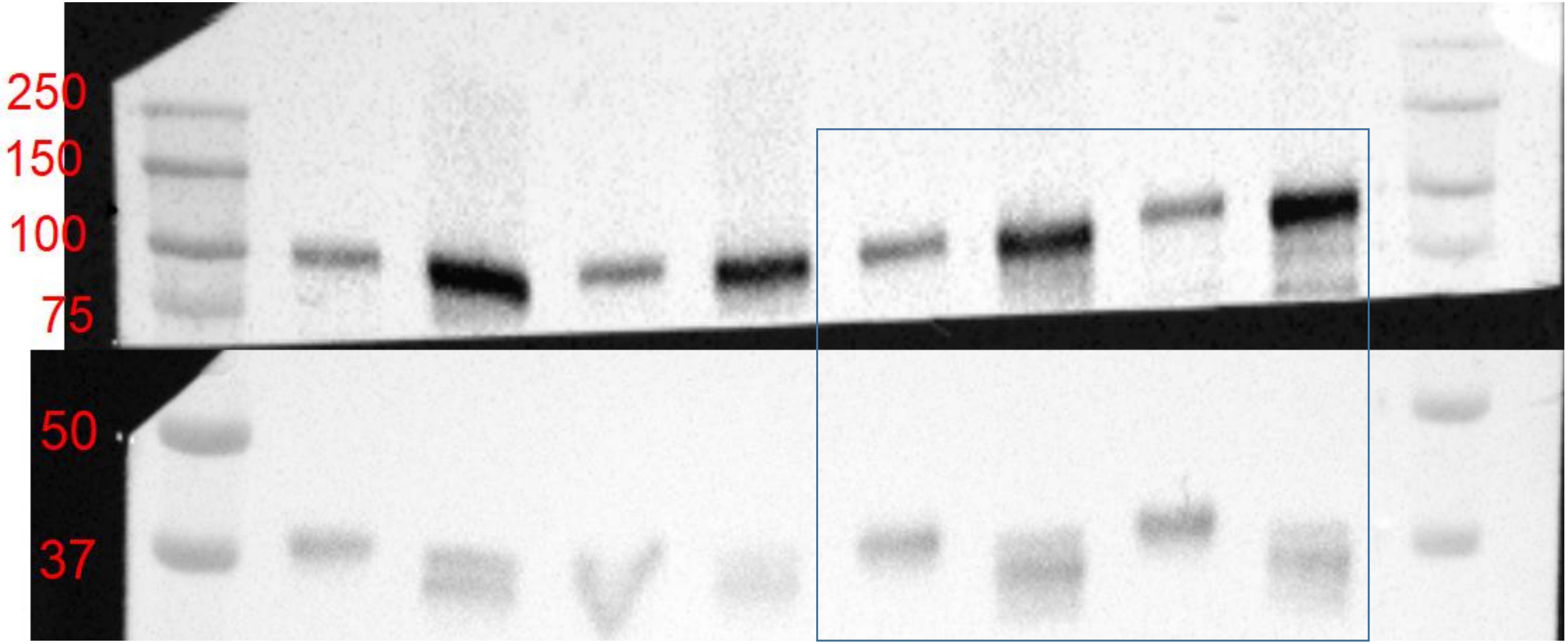

Suppl Fig 5

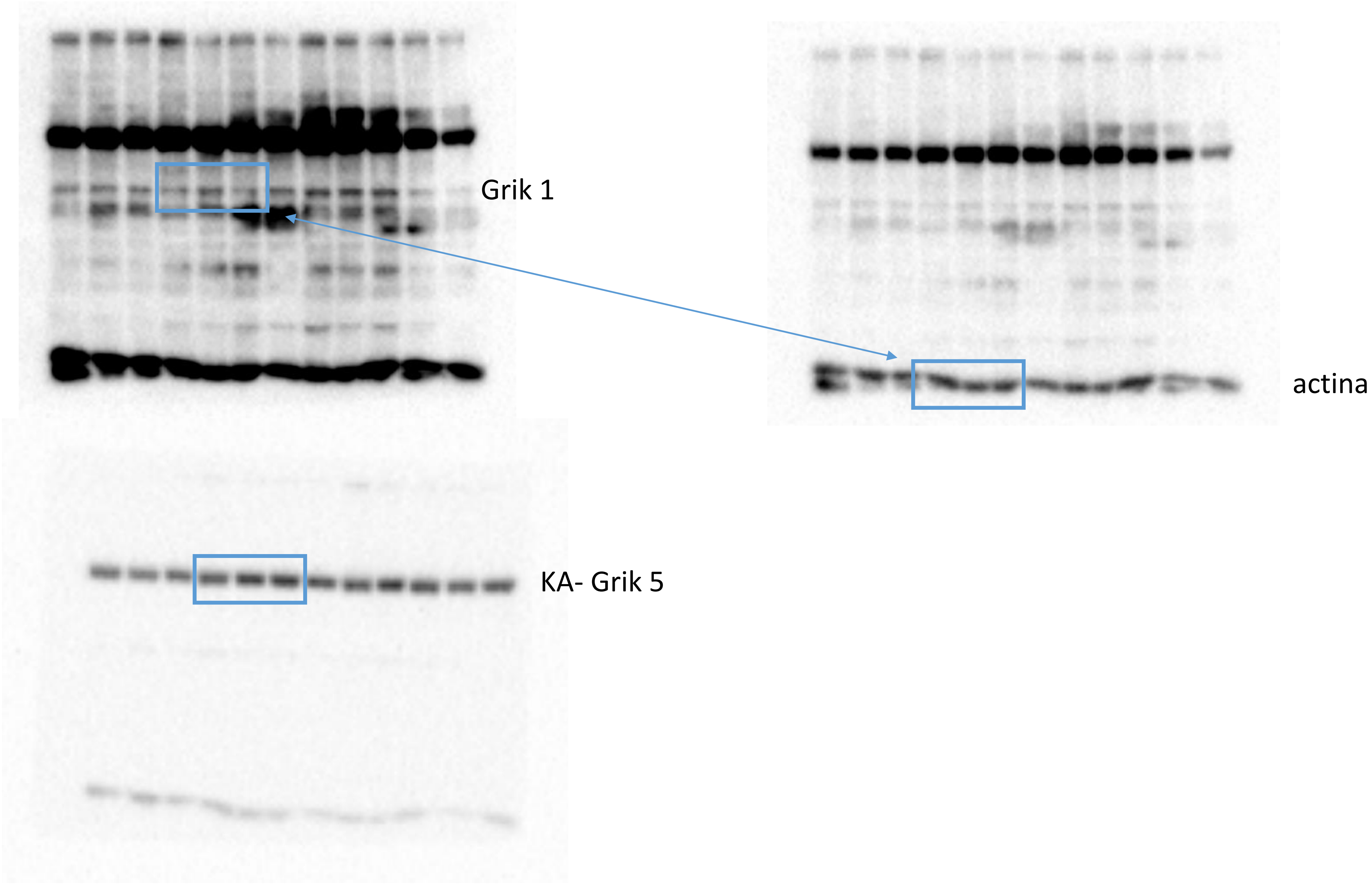

Supplement: Supplementary file 9 — UNCROPPED original western blots [file 41419_2022_5038_MOESM9_ESM.pdf]
